# Evaluating Purifying Selection in the Mitochondrial DNA of Various Mammalian Species

**DOI:** 10.1371/journal.pone.0058993

**Published:** 2013-03-22

**Authors:** Pedro Soares, Diogo Abrantes, Teresa Rito, Noel Thomson, Predrag Radivojac, Biao Li, Vincent Macaulay, David C. Samuels, Luísa Pereira

**Affiliations:** 1 Instituto de Patologia e Imunologia Molecular da Universidade do Porto (IPATIMUP), Porto, Portugal; 2 Department of Statistics, University of Glasgow, Glasgow, United Kingdom; 3 School of Informatics and Computing, Indiana University, Bloomington, Indiana, United States of America; 4 Center for Human Genetics Research, Department of Molecular Physiology and Biophysics, Vanderbilt University Medical Center, Nashville, Tennesee, United States of America; 5 Faculdade de Medicina da Universidade do Porto, Porto, Portugal; University of Florence, Italy

## Abstract

Mitochondrial DNA (mtDNA), the circular DNA molecule inside the mitochondria of all eukaryotic cells, has been shown to be under the effect of purifying selection in several species. Traditional testing of purifying selection has been based simply on ratios of nonsynonymous to synonymous mutations, without considering the relative age of each mutation, which can be determined by phylogenetic analysis of this non-recombining molecule. The incorporation of a mutation time-ordering from phylogeny and of predicted pathogenicity scores for nonsynonymous mutations allow a quantitative evaluation of the effects of purifying selection in human mtDNA. Here, by using this additional information, we show that purifying selection undoubtedly acts upon the mtDNA of other mammalian species/genera, namely *Bos sp.*, *Canis lupus*, *Mus musculus*, *Orcinus orca*, *Pan sp.* and *Sus scrofa*. The effects of purifying selection were comparable in all species, leading to a significant major proportion of nonsynonymous variants with higher pathogenicity scores in the younger branches of the tree. We also derive recalibrated mutation rates for age estimates of ancestors of these various species and proposed a correction curve in order to take into account the effects of selection. Understanding this selection is fundamental to evolutionary studies and to the identification of deleterious mutations.

## Introduction

In the last few years, it has been suggested that mitochondrial DNA (mtDNA) variation in humans and other species does not fit the expectations of the neutral model, and is instead under the influence of purifying selection (as reviewed in [1], [2]).

In the nineties some authors [3] compared the sequences of the gene *MT-ND3* across 61 humans, five chimpanzees, and one gorilla and observed that the ratio of replacement to silent nucleotide substitutions was higher within humans and within chimpanzees than when comparing the two species. This result was later confirmed in 17 out of 25 examined animal species [4] and was attributed to a substantial number of mildly deleterious amino acid mutations that rarely become fixed in the population. Other authors [5] also detected a deviation from the neutrality model in animal mtDNA using the McDonald/Kreitman test.

Reports on the effect of purifying selection against nonsynonymous mutations in mtDNA were also described when comparing recent and ancient mtDNA samples in Adélie penguins [6]. Subramanian and Lambert [7] detected similar synonymous mutation rates when comparing a clade within the human mtDNA tree and the split between the human and chimpanzee but the non-synonymous rate was 9–15 times higher in the former. It is possible that up to 80% of the observed amino-acid replacements detected in humans are slightly deleterious [8]. Purifying selection acting on slightly deleterious changes (not only non-synonymous mutations) is probably the major force causing this apparent time-dependent mutation rate (faster mutation rates in short time-frames and slower rates at long-term estimates) [1], [2] but some other factors might play a role. One factor is saturation that would hide mutations in long-term phylogenetic relationships and apparently slow the mutation rate. Another factor is sequencing error that generates extra incorrect mutations in closely-related sequences artificially raising the mutation rates [9].

Peterson and Masel [10] also detected elevated Ka/Ks (ratio of non-synonymous *vs.* synonymous mutations) within a recently established population against long-term Ka/Ks but they propose ancestral polymorphisms as the main cause for the observed rate elevation. One point that suggests that this factor might not be the most important cause for time dependent rates is the fact that, at least for mtDNA, the higher estimated mutation rates are obtained in pedigree studies where ancestral polymorphisms are not an issue [11], [12]. Additionally mutation rate estimates obtained from ancient DNA sequences that are calibrated by the age of the sample and not the age of the node also report faster short-term mutation rates [10], [13]–[16].

Human mtDNA has been a primary object of study and has played an important role in the overall discussion about time-dependent mutation rates and the effect of purifying selection. In a study using 104 worldwide human mtDNA complete sequences [17] the observed higher frequency of non-synonymous mutations in the mtDNA genomes of people from the Arctic and temperate zones when compared with Africans suggested that climate was a positive selective force that shaped human mtDNA variation. This claim has been refuted by others [18]–[21], who showed that selection has acted similarly across the human mtDNA tree. The higher frequency of non-synonymous mutations observed by Mishmar and colleagues [17] is a general feature of the younger branches of the human mtDNA tree [19]. Many of these studies used a phylogenetic approach but they mostly compared haplogroups observed in different geographic regions [17], [19], [20] or compared terminal branches to internal branches of the tree [18], [22]. The haplogroup methods suffer from a problem of mixing lineages with diverse times of emergence, whereas the branch comparison method suffers from a similar problem of combining branches that can differ by almost 200,000 years [23] in their time of formation.

The hierarchical structure of the human mtDNA tree allows one to measure the depth of any node in the tree and to establish a relative depth of any mutation, avoiding any ambiguous classification. Both Soares et al. [24] and Pereira et al. [21] used the ρ statistic which measures the average number of sites differing between a set of sequences and their common ancestor [25]. Another advance of the Pereira et al [21] analysis is that non-synonymous mutations are additionally defined by a quantitative pathogenicity score (ranging from 0, neutral, to 1, probably highly pathogenic), avoiding the standard practice of lumping together all nonsynonymous variants including quasi-neutral substitutions of amino acids with similar physical and chemical properties [26], [27] as equally deleterious. The tested score was MutPred [28], which builds on the well-established SIFT method [27] and is based upon the protein sequence, structural features, and comparison between functional sites in putatively neutral variants and reported pathogenic mutations. A comparison of MutPred scores with the scores from a second pathogenicity predictor algorithm (denominated SNPs&GO) [29] in somatic mtDNA mutations in cancer [30] confirmed its reliability, as was previously shown for nuclear mutations [31]. Pereira et al. [21] concluded that protein variants with high pathogenicity scores were significantly rarer in the older branches of the tree compared to nonsynonymous variants with low pathogenicity scores.

One important aspect of the phylogenetic approach is the inclusion of a time frame based on the molecular clock; however, for many years the clock was based on a linear relation between the accumulation of substitutions and time (e.g. [17]). Because selection is overlooked, the higher proportion of nonsynonymous mutations in the younger branches leads to an overestimation of their age. Several suggestions have been advanced in order to resolve this issue. Kivisild et al. [19] suggested that only the third codon positions (mostly synonymous variations) should be used for estimation of the mutation rate, but these sites represent only a limited portion of the molecule, leading to very large confidence intervals in the results. Other authors used a Bayesian recalibration assuming intraspecific calibration points (based on archaeological information) but if many internal calibration points are debatable in humans [32]–[37], they are generally unknown for many species.

Ho and Larson [38] suggested as an alternative the estimate of a curve in order to assess mutation rates needed for the timescale required. Henn and colleagues [39] characterized the time-dependent rate curve based on the correlation between the age of a several clades and specific events in human evolution but, as mentioned above, the timing of many of these events is debatable. Soares et al. [24] developed a calibration curve for the human mtDNA mutation rate that corrects for its time dependency, including the modest effect of selection. The authors assessed the variation of different classes of mutation at different time depths (defined by ρ statistics) for a global mtDNA tree inferred from ∼2,000 complete genomes. This allowed them to estimate the fraction of synonymous mutations across all mutations over time (if synonymous mutations accumulate almost linearly with time, the other classes do not due to the purifying selection); this fraction showed a tendency to an asymptote when a Gompertz function was fitted to the data. They also showed that these deviations could be better explained by the effect of purifying selection than by saturation, said to occur mainly in the control region [19]. The equation of the fitted curve was then used to correct the molecular clock recalibrated by the time split from an outgroup (the *Homo*-*Pa*n split).

In the work reported here, we aim to:

- Characterize the effect of purifying selection in the mtDNA of several mammals, including species with different biology and environments. We use quantitative measures of variation over time which allow a direct comparison of selection between species;
- Estimate the mutation rate for the protein-coding mtDNA and apply a correction for the effect of purifying selection in several mammalian species as has been successfully implemented and widely used in human mtDNA. We also recalibrate the mammalian tree in order to obtain split times between species where the fossil evidence is absent.

## Materials and Methods

### Sequences

We are limited to those species/genera for which we can infer a reliable intraspecific phylogenetic tree based on the available sequence data. These are several species of *Bos* (including *B. taurus*, *B. grunniens, B. indicus, B. javanicus, B. primigenius* and the closely related *Bison bison* although it is classified in a different genus), *Canis lupus* (including *C. lupus lupus* and *C. lupus familiaris*), *Mus musculus*, *Orcinus orca*, *Pan* (*P. troglodytes* and *P. paniscus*) and *Sus scrofa*. The sequences used in this work were extracted from the GenBank database [40] using the Geneious software [41] and are reported in Table S1. Their alignment was performed by the Clustal W algorithm implemented in BioEdit software [42] versus a reference sequence, for which we followed the sequence reported in RefSeq database [43] (NC_006853 for *Bos taurus*; NC_002008 for *Canis lupus familiaris*; NC_005089 for *Mus musculus*; NC_014682 for *Orcinus orca*; NC_001643 for *Pan troglodytes*; NC_000845 for *Sus scrofa*). In the special case of *Pan troglodytes*, the reference sequence was not included in the final analysis due to the detection of errors in this sequence, resulting most probably from the mixing of fragments between samples, but its numbering system was maintained to allow continuity with the previous literature. As mtDNA is circular, the initial sequence position is arbitrary and some of the released sequences do not follow the consensus sequence start position. These cases were re-oriented using the CSA software [44], but maintaining the original numbering of the reference sequence for each species.

The aligned sequences were input in the mtDNA-GeneSyn software [45] to identify the polymorphic positions. This software also allows the user to extract the polymorphic positions into the input format for the Network software [46], which we used in a first step to detect possible errors in the sequences available in GenBank. For instance, the most common error we observed was the exchange of fragments between samples, which is identifiable in networks by recurrence involving many polymorphisms closely located in the mtDNA genome. The final number of sequences considered per species/genus was: 280 for *Bos* (176 *B. taurus*, 67 *B. grunniens,* 3 *B. indicus,* 1 *B. javanicus,* 1 *B. primigenius* and 32 *Bison bison*), 262 *Canis lupus* (including 7 *C. lupus lupus* and 255 *C. lupus familiaris*), 77 *Mus musculus*, 63 *Orcinus orca*, 55 *Pan* (33 *P. troglodytes* and 22 *P. paniscus*) and 69 *Sus scrofa*.

Some species have motifs repeated several times in tandem in the control region (for instance *Canis lupus* has a 10 bp motif [47]). As some authors do not sequence these regions, and the tandem repetitive motifs obey a step-wise mutation model, we did not include these repetitive regions.

We also performed alignments of the protein-coding region in 320 mammalian species (Table S2). We limited this alignment to that region because we saw that the size and sequence of the control region diverged greatly between species, and even the rRNA regions differed considerably in inter-species comparisons. At the same time, although probably not a major problem in intraspecific phylogenies, this limitation eliminated issues like saturation in the control region and non-independence of mutations when incorporated in secondary structures in RNAs and even the control region [48]. We extracted individually the 13 protein-coding genes by using the mtDNA-GeneExtractor tool [49], and aligned them through the Clustal W algorithm. We also constructed alignments for the 13 protein-coding genes in each of the species studied here and, for comparison purposes, also in human mtDNA using the sequences from the African tree [50] (Table S3). We limited the human analysis to the African tree because we wanted to have a similar number of individuals to the other species trees and that is the most diverse group of the human species. This choice does not bias the results as we previously showed that the effect of purifying selection acts in a similar way in the African, European and Asian trees [21].

### Phylogenetic Reconstruction

We constructed phylogenetic trees based on the application of the reduced-median algorithm [46], from the Network software, to the observed substitutions in the complete mtDNA genomes for each species. This algorithm follows the rules of the most parsimonious model of evolution. It also facilitates quality control by allowing the detection of reticulations that most probably reflect mixing of fragments between samples during lab work. The basic phylogeny was also tested using the MrBayes software [51].

We then annotated the branches, identifying synonymous and nonsynonymous polymorphisms. We used this information to calculate the ρ statistic (the average number of sites differing between a set of sequences and a specified common ancestor) for each node of the tree in two ways: an overall protein-coding rho, by considering all mutations in the protein-coding genes; and a synonymous rho, by only counting synonymous mutations. Because polymorphisms are associated with branches and ρ values are properties of nodes, a choice between the ρ values of the upper or lower nodes on the branch (or an average representing the center of the branch) must be made. We used the ρ of the lower end of the branch, which provides a lower bound on the depth of the polymorphism in the tree. Considering that there is extensive evidence for purifying selection on mtDNA variants, it is likely that many of the observed variants defining a branch formed recently, also leading us to choose the lower node rho. Each polymorphism had two associated ρ values (an overall protein-coding ρ and a synonymous rho) for each species. Recurrent polymorphisms appearing in many branches of the tree were included in the analysis separately as many times as they independently arose.

### Pathogenicity Score and Evaluation of Purifying Selection

For each species, we used the reference sequence to construct artificial DNA sequences for every possible transition and transversion. These artificial genomes were then analyzed in the mtDNA-GeneSyn software [45] to identify and classify all of polymorphisms which could occur in the mtDNA of each species. From these, we extracted the list of all possible nonsynonymous substitutions (not considering the ones involving stop codons) and used them for the MutPred pathogenicity score analysis [28], reported in Supporting Information S1. MutPred is a measure that probabilistically estimates the impact of an amino-acid change in the protein based upon the protein sequence, structural features, and comparison between functional sites in putatively neutral variants and reported pathogenic mutations. The pathogenicity prediction method was trained using solely human molecular and genetic data but since the dominant feature of MutPred is sequence conservation such functions should be transferrable across species. The pathogenicity scores used in a previous analysis in humans [21] were calculated using version 1.1 of the MutPred software. The pathogenicity scores used in this analysis in all species were calculated using an updated version of the software, version 1.2. For consistency, the pathogenicity scores for the human dataset were recalculated using version 1.2 of the MutPred software, and are thus slightly different from the values previously reported [21]. We provide on request lists of all possible single amino acid variants for the various species mtDNA-encoded proteins with their predicted pathogenicity scores as a tool for assessing novel protein variants reported in other works.

The MutPred pathogenicity scores of the observed nonsynonymous mutations in each species were extracted from the total list, and these values together with the ρ values were used for statistical evaluation of purifying selection. In order to check the distribution of pathogenic mutations across the tree we compared the average MutPred pathogenicity scores for variants with ρ values lower than 4 (recent variations) to those variants with ρ higher than 4 (older variations). We also compared the average ρ value for the set of variants with Mutpred scores >0.7 (higher pathogenicity) to variants with MutPred scores <0.7 (lower pathogenicity). All comparisons of mean values were calculated in Origin 7 (www.originlab.com), by using two-tailed t-tests assuming unequal variances. A selection function for the nonsynonymous polymorphisms was calculated by dividing the distribution of scores for observed nonsynonymous polymorphisms, P_obs_, by the distribution of scores for all possible nonsynonymous polymorphisms, P_poss_. An exponential curve of the form 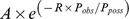, where R is defined as the pathogenicity selection constant was fit to the data. The values of the parameters A and R were set by a nonlinear curve fit carried out in Origin 7.

### Curves of Correction for Purifying Selection

For each node 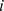 in the tree, the ratio of the synonymous variant 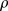 to the overall variant 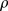 (the “synonymous 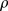 proportion”: 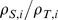) was plotted against the overall 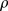 (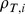). A Gompertz function was used to model the expected synonymous 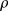 proportion 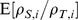 in terms of the overall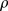, the curve-fitting performed using non-linear least squares in R [52], as described by Soares et al. [24]. Because the 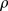 values at different nodes are correlated (due to the nesting in the tree), and also because the variance changes with overall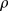, we adopted a strategy of agglomerating nodes to ameliorate these effects, as follows. We sorted and reindexed nodes by decreasing overall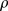. Neighboring points in the sorted list should have approximately the same synonymous 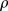 proportion, at least in expectation. Thus 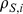 (as well as 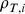) was accumulated up the list until an estimate of the variance of 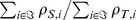
(for 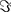 the set of nodes agglomerated) was reduced below some user-defined threshold 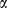, when a new averaged data pair 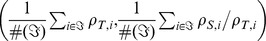 was produced and a fresh accumulation of nodes started. This process was repeated until all the nodes were used up. Sensitivity of the fitted model to 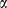 was not fully explored, but the results of limited investigation of the effect of changing 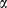 were encouraging.

### Calibration Points

In order to define calibrated outgroups for each species/genera set of sequences to estimate mutation rates, we performed a phylogenetic analysis on the 320 mammalian species (Table S2) for the protein-coding genes. We obtained the phylogeny through a Bayesian analysis, by using the MrBayes software [51], assuming a HKY85 model of DNA evolution (two rate parameters). The resulting MrBayes phylogeny, in the Newick format, was visualized in the FigTree software (http://tree.bio.ed.ac.uk/software/figtree).

We compared the phylogeny obtained with the mammal taxonomy and the available fossil record in order to establish which taxonomic groups were monophyletic in the analysis and, in this way, which groups could be reliable calibration points. We checked the reconstructed mammalian tree based on mtDNA diversity and focused on branches that were reliably supported by both the known taxonomy and the mtDNA phylogenetic reconstruction.

We then used BEAST [53] to calibrate the mammalian tree using a relaxed clock (which is an important feature considering the wide range of species and probable large range of mutation rates), the HKY85 model of molecular evolution and gamma distributed rates (10 gamma categories). The analysis ran for a total of 200,000,000 states in the Markov chain. We calibrated the analysis at several internal nodes by using fossil evidence [54] to inform their prior age distributions. Fossil evidence is also displayed in the “date a clade” website (http://www.fossilrecord.net/dateaclade/index.html). These are reported in Table S4. All the hypothetical calibration points were presented as minimal and maximum constraints for a given split in the tree. Minimum and maximum constraints are generally very conservative so we opted for a normal distribution where the higher probability of the split will take place at intermediate dates of the distribution [55]. Given this, we established the middle point as the point estimate and fixed the 95% confidence interval of a normal distribution to fit the maximum and minimum constraints. In this way, we can obtain a reanalysis of all the point estimates of each of these calibration points based on the tree and all the other priors and at the same time to estimate an age for splits where no fossil date is available. These were mainly splits between the studied species and close relatives, which will be essential for determining specific substitution rates.

### Estimating Mutation Rates

Mutation rates for each of the species were calculated using a maximum likelihood (ML) approach with the PAML software [56], and the calibration points estimated above (as presented in the Results section). Mutation rates were calculated for overall protein-coding genes and for synonymous mutations. The synonymous mutation rate was calculated with CODEML after readjusting the ND6 gene to the same reading direction as the other 12 genes, deleting the termination codon, and changing all the nonsynonymous mutations into a possible ancestral state leaving only the synonymous mutations in the alignment. The rate of synonymous mutations was estimated based on the mammalian mtDNA genetic code and was calculated as the number of synonymous mutations per codon and not per position. The HKY85 model of nucleotide evolution (a model that distinguishes two substitution rate parameters - transitions and transversions) was used with gamma-distributed rates (approximated to 32 gamma categories). Gamma categories might be excessive in both the ML and Bayesian analyses but we opted for being conservative in this aspect. Analyses assuming the GTR model of nucleotide evolution (a model that distinguishes six substitution rate parameters) and not assuming a clock were run, in order to perform likelihood ratio tests (LRT) [57] comparing these two models of evolution and the reliability of a molecular clock.

The ρ values based on protein-coding genes and synonymous mutations calculated above within the trees of the different mammals were used to calculate age estimates of each node based on the two clocks and both ages were compared after the correction of the protein-coding estimates with the Gompertz curve.

We additionally estimated the interspecific mutation rates using the BEAST software and the same datasets described for the PAML analysis. In order to test different models we calculated Bayes factors [58] in Tracer (http://beast.bio.ed.ac.uk/Tracer) comparing the HKY85 and the GTR models. We also compared the performance between a strict and a relaxed clock model, since intra-specific mutation rate variation between lineages is a possibility.

## Results

### Purifying Selection

**Figure 1:**
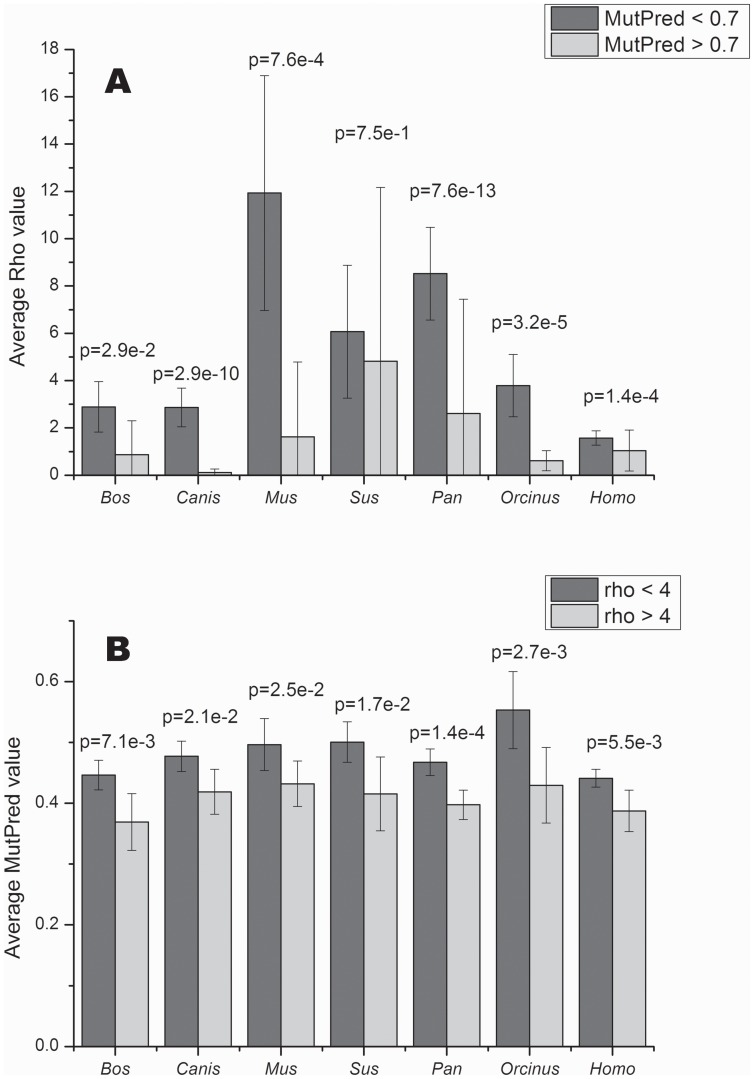
Comparison between average pathogenicity scores and tree depth. (A) shows a comparison of the average ρ values for nonsynonymous substitutions in the trees for pathogenicity scores lower and higher than 0.7; (B) shows the average pathogenicity scores for all nonsynonymous substitutions occurring in the trees binned by the ρ value (<0.4 and >0.4). P-values are from two-tailed t-tests.

The pathogenicity scores for all nonsynonymous substitutions occurring in all mammalian species analyzed (216 for *Bos*, 240 for *Canis*, 114 for *Mus*, 66 for *Orcinus*, 330 for *Pan* and 132 for *Sus*) displayed a trend of decreasing ρ value as the pathogenicity score increases. The rapid drop in average ρ for pathogenicity scores greater than 0.6 to 0.7 described in humans (634 substitutions; [21]), is also observed in these species. Based on this observation, we plotted the average ρ for two classes of pathogenicity scores (<0.7 and >0.7). The average ρ was statistically significantly lower for the class with higher pathogenicity score in all species (except for *Sus*, where the difference did not reach statistical significance due to the high standard errors; Figure 1A). This is consistent with selection against these particular non-synonymous variants. Conversely, variants in the younger branches of the phylogenetic trees (ρ <4) had statistically significantly higher average pathogenicity scores compared to the variants in the older part of the trees (ρ >4) (Figure 1B).

**Figure 2:**
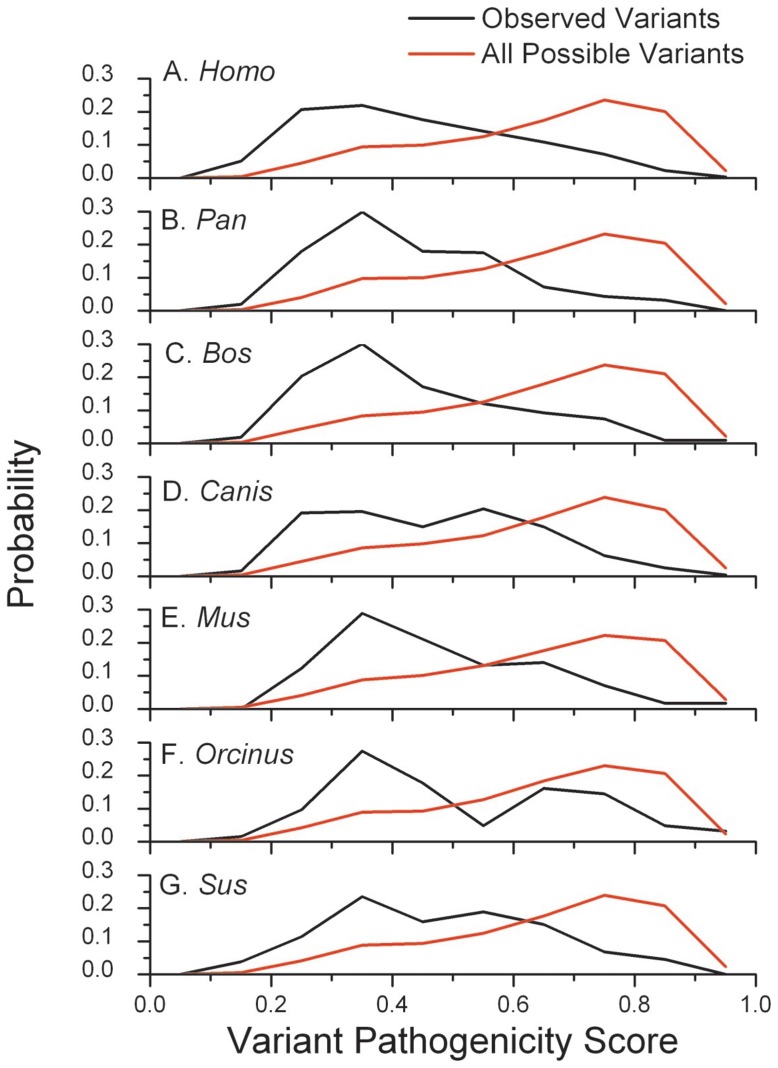
Probability distributions of the observed nonsynonymous pathogenicity scores for each species compared against the probability distributions of all possible substitutions from the reference sequence.

As the observed nonsynonymous substitutions in the trees are only a small fraction of all the possible nonsynonymous variants that could occur through a single nucleotide change from each species’ reference sequence, we compared the distributions of pathogenicity scores between observed and all possible nonsynonymous substitutions (Figure 2). As observed previously in humans [21] (and again reported in Figure 2), in each species the set of all possible substitutions is strongly skewed toward higher pathogenicity scores, indicating that a large fraction of variants are deleterious. In all but one species, the variants with MutPred pathogenicity scores in the range 0.3–0.4 (low pathogenicity) had the highest observed probability. The exception is *Canis* where the probability distribution of the observed variants is quite broad with the highest probability in the 0.5–0.6 class. This could reflect a higher amount of slightly deleterious mutations in the dog tree. The distributions for the observed substitutions have a much lower average pathogenicity score than the set of all possible substitutions (comparisons in each species are statistically significant; p<10^−5^). As can be seen in Figure 2, the distribution of the pathogenicity values for the set of all possible variants is quite similar across these species, and the average value of this distribution is very close in all species, between 0.640–0.646.

**Figure 3:**
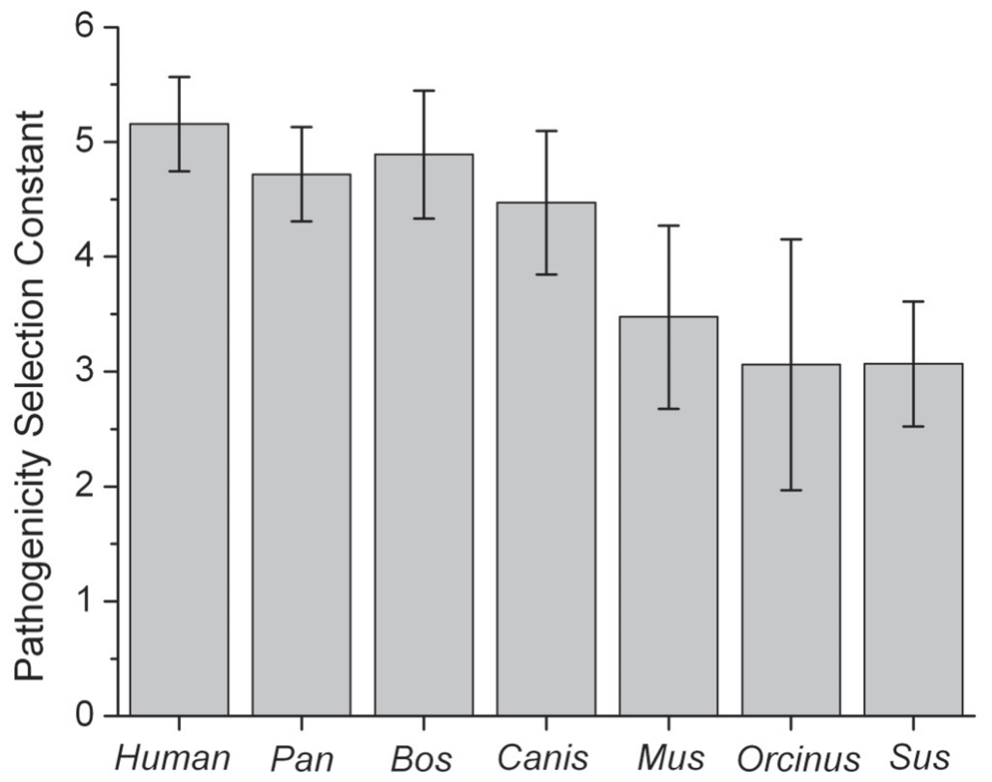
The pathogenicity selection constant R for each group of mammalian species. The values and their error bars were calculated by a fit of a decaying exponential curve to the ratios of the probabilities plotted in Figure 5. Higher values of R represent greater purifying selection. For the exponential fits, see Figure 1.

One method of assessing how strongly the purifying selection varies with the pathogenicity score of the variation is to take a simple ratio of the distributions plotted in Figure 2, the probabilities of the observed variants, P_observed_, divided by the probabilities of all possible variants, P_allpossible_, (Figure S1). Previously it was shown that in humans this measure decreased exponentially as the pathogenicity score of the variant increased [21], following the formula (P_observed_/P_allpossible_) = A exp (−Rg), where g is the pathogenicity score and A and R are constants. The constant R defines how quickly the exponential decrease in the observed variants probability occurs, with higher values corresponding to stronger purifying selection. We refer to this constant R as the pathogenicity selection constant. Our analysis shows that all mammalian species examined here also showed an exponential decrease in this ratio of probabilities (Figure S1). Furthermore, the pathogenicity selection constant R has similar values across this group of mammals (Figure 3), with the *Sus*, *Orcinus* and *Mus* groups presenting the lowest pathogenicity selection constant R calculated, raising the possibility of a slightly weaker effect of purifying selection in these species.

The comparison of the proportion of synonymous mutations and non-synonymous mutations across the trees of the different species is a complementary and independent assessment of the effect of purifying selection compared to the MutPred pathogenicity score analysis described above. While the latter shows that lineages containing deleterious mutations do not survive to become older branches, this analysis shows that the proportion of non-synonymous variants decreases in the older branches, as expected.

**Figure 4:**
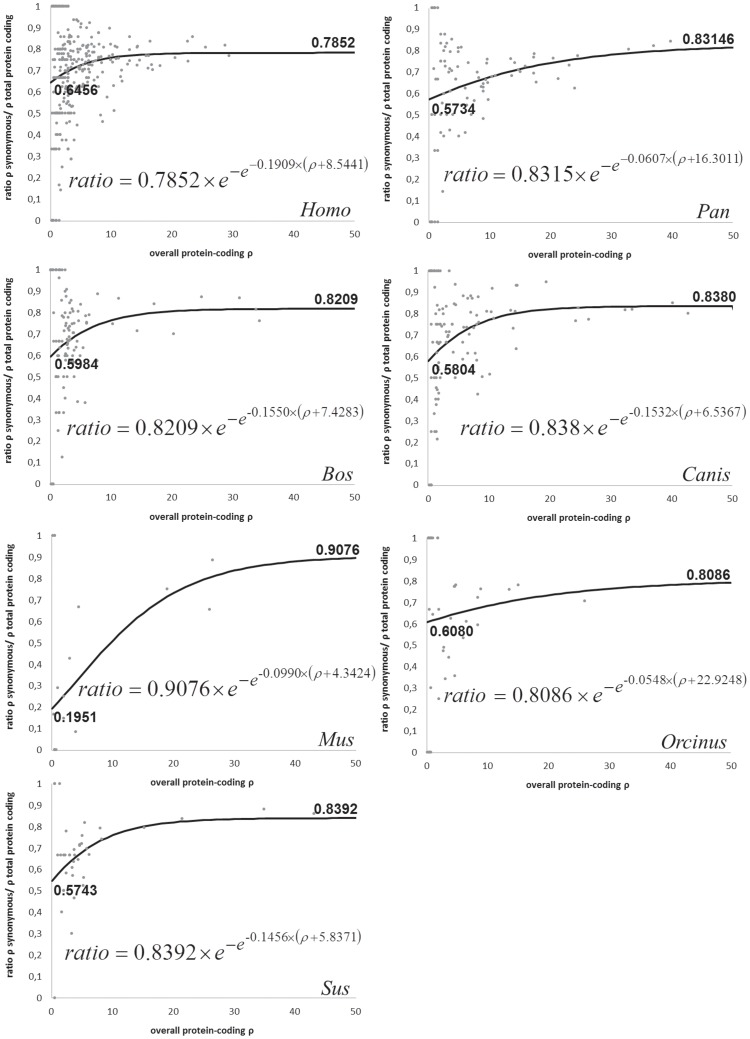
Gompertz function (black lines) of the relation between ρ value and ratio of ρ synonymous to total protein-coding ρ obtained from the data points of the different species (in grey). Formulas are displayed in the graphics.

Following the approach used before [24] we fitted a Gompertz function, which has an initial fast increase and then continuously tends to an asymptotic value, representing the theoretical proportion of synonymous variants in the total fixable variants in the interspecific phylogeny (% synps, percentage of synonymous post-effect of selection in [24]). The results for all the species are shown in Figure 4. The curve fitting worked well for the human, dog and cattle data (the species with the largest datasets) and we obtained curves with lower confidence for the killer whale, pig and chimpanzees. A curve for the mouse was obtained with very low confidence given the fact that it was based on a much lower number of nodes.

### Protein-coding Mutation Rates Estimates

**Figure 5:**
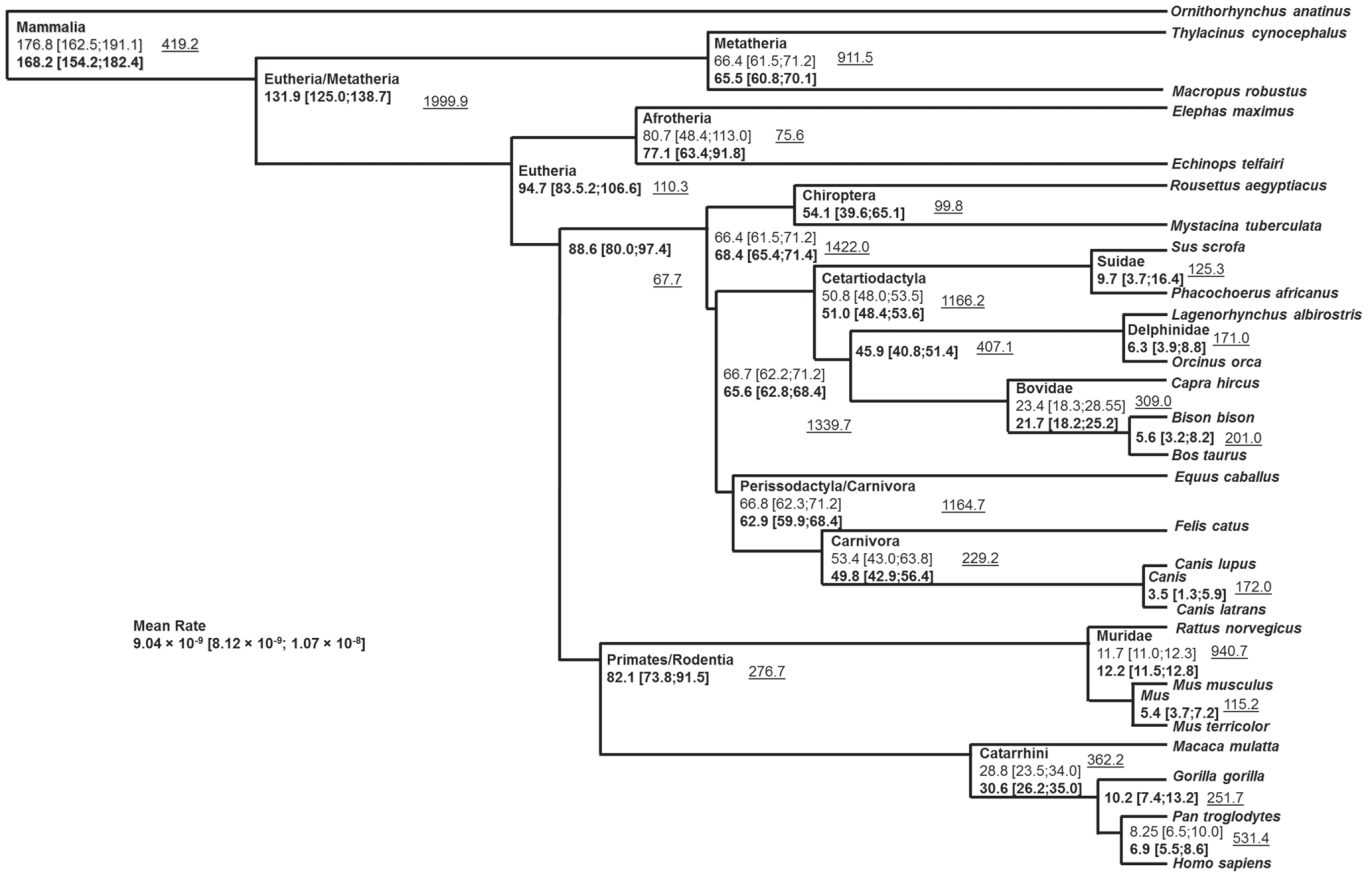
Schematic tree displaying the divergence between groups as obtained from the BEAST analysis. Species are representatives of the displayed branches. Age (in millions of years) and 95% confidence intervals displayed in regular font are the used priors. Ages in bold represented the posterior values obtained in the analysis. The underlined value indicates the effective sample size of each estimate.

Based on a set of mtDNA reference sequences for 320 mammalian species (Table S2), we created an mtDNA mammalian tree in BEAST containing several priors (calibration points). As this high number of sequences exceeds the advised capacity of that software (200 sequences is the limit suggested by the authors), we excluded closely related species following the Bayesian phylogenetic reconstruction with MrBayes [51] for a final dataset of 177 sequences (Table S5). The priors were selected on the basis of available calibration points (or intervals) and following the phylogenetic reconstruction with MrBayes where we checked that those groups were monophyletic for the mtDNA. Priors are described in Table S4 and Figure 5 and the results of the BEAST analysis (posterior distribution) for these and for other points of interest are shown in Figure 5. All the posterior estimates for the priors we stipulated were within the minimum and maximum constraints defined by the fossil record [54]. The results provided an overall mutation rate for the mtDNA protein-coding region of mammals of 9.04×10^−9^ substitutions per nucleotide per year. As mentioned before, we did not aim to provide a definitive mammalian phylogeny using mtDNA, so taxonomic groups that were monophyletic in the MrBayes analysis (and where paleontological dates were available) were used as priors. It is worth mentioning the main difference in the primary splits of the tree between the trees obtained in MrBayes and BEAST and the accepted taxonomy. In the MrBayes reconstruction the order Eulipotyphla appears as a sister clade to all the remaining Eutheria while in BEAST this position is taken by the superorder Xenarthra.

We ran maximum likelihood (ML) analyses using PAML on each of the species rooted by their closest relatives, both using the complete variation in the protein-coding genes as well as an alignment containing only the synonymous mutations. The latter was run with CODEML taking into account the vertebrate mtDNA genetic code in order to estimate the number of synonymous mutations per synonymous possibilities in the present codons. We also used BEAST to calculate protein-coding mutation rates using both a strict and a relaxed clock. We obtained mutation rates using the calibration points indicated in Figure 5. Mutation rates obtained using PAML are displayed in Table 1 while mutation rates obtained from BEAST are displayed in Table 2. Mutation rates calculated using PAML or BEAST are similar in all the analyses with about 10% differences between analyses for the same species. The case where this value differs the most is when using a relaxed clock (in BEAST) against a strict clock (both from BEAST and PAML) in *Mus musculus.* In the different analyses, *Mus musculus* is the species that displayed the most divergent mutation rate with an mtDNA protein-coding mutation rate more than twice as fast as the second fastest estimated rate in the analysis (*Homo/Pan*).

**Table 1:** Synonymous and non-synonymous substitution rates obtained using maximum likelihood for several species or group of species.

	Likelihood ratio test (p-value)		Protein-coding mutation rate		Synonymous mutation rate	
Species	HKY85 *vs.* REV nucleotide substitution models	Clock *vs.* No Clock model	Substitutions per nucleotide per year estimated from PAML (and standard error)	Average number of years for a mutation to happen	Synonymous mutations per codon per year (andstandard error)	Average number of years for a synonymous mutation to happen
*Homo sapiens/Pan troglodytes/Pan paniscus*	0.73	0.10	1.29×10^−8^ (4.99×10^−10^)	6790	3.27 ×10^−8^ (5.55×10^−9^)	8027
*Canis lupus*	0.07	0.63	9.68×10^−9^ (4.52×10^−10^)	9026	2.45×10^−8^ (1.06×10^−9^)	10684
*Bos taurus/Bos grunniens/Bison bison*	0.08	0.99	9.00×10^−9^ (3.66×10^−10^)	9708	2.12×10^−8^ (8.33×10^−10^)	12393
*Sus scrofa*	0.02	0.06	1.18×10^−8^ (5.87×10^−10^)	7424	2.90×10^−8^ (4.90×10^−9^)	8742
*Orcinus orca*	0.08	0.99	1.22×10^−8^ (1.49×10^−9^)	7143	2.66×10^−8^ (8.69×10^−10^)	9842
*Mus musculus*	1.2×10 ^−6^	0.17	2.78×10^−8^ (2.13×10^−9^)	3035	7.72×10^−8^ (3.91×10^−9^)	3396

P-values of likelihood ratio tests (LRT) comparing DNA evolution models and the use of a molecular clock were calculated. Significant P-values are underlined.

**Table 2:** Protein-coding substitution rates obtained using Bayesian analysis for several species or group of species.

	Protein-coding mutation rate (Substitutions per nucleotide per year estimated from PAML (and Standard Error)				Bayes Factors			
Species	Strict Clock with HKY85 model	Strict Clock with REV model	Relaxed Clock with HKY85 model	Relaxed Clock withREV model	HKY85 *vs.* REV in strict clock	HKY85 *vs.* REV in relaxed clock	Strict *vs.* relaxed clock using HKY85 model	Strict *vs.* relaxed clock using REV model
*Homo sapiens/Pan troglodytes/Pan paniscus*	1.21×10^−8^(6.44×10^−10^)	1.22×10^−8^(6.91×10^−10^)	1.22×10^−8^(4.63×10^−10^)	1.21×10^−8^(5.49×10^−10^)	30.4 (strong)	30.5 (strong)	2.1 (weak)	1.8 (weak)
*Canis lupus*	8.57×10^−9^(5.26×10^−10^)	8.52×10^−9^(5.10×10^−10^)	8.63×10^−9^(7.15×10^−10^)	8.40×10^−9^(4.13×10^−10^)	4.4 (positive)	5.1 (positive)	1.4 (weak)	2.1 (weak)
*Bos taurus/Bos grunniens/Bison bison*	8.65×10^−9^(2.85×10^−10^)	8.65×10^−9^(3.15×10^−10^)	9.26×10^−9^(5.61×10^−10^)	9.99×10^−9^(5.62×10^−10^)	1.9 (weak)	9.2 (positive)	22.7 (strong)	30.9 (strong)
*Sus scrofa*	1.03×10^−8^(4.44×10^−10^)	1.02×10^−8^(3.78×10^−10^)	9.81×10^−9^(6.88×10^−10^)	9.62×10^−9^(5.67×10^−10^)	1.8 (weak)	2.0 (weak)	1.9 (weak)	2.4 (weak)
*Orcinus orca*	1.10×10^−8^(9.82×10^−10^)	1.12×10^−8^(1.10×10^−9^)	9.31×10^−9^(6.58×10^−10^)	9.17×10^−9^(5.92×10^−10^)	0.8 (none)	1.4 (weak)	6 (positive)	6.5 (positive)
*Mus musculus*	2.59×10^−8^(1.83×10^−9^)	2.66×10^−8^(2.75×10^−9^)	1.97×10^−8^(1.94×10^−9^)	2.03×10^−8^(1.81×10^−9^)	16.0 (positive)	16.1 (positive)	13.5 (positive)	13.6 (positive)

Bayes factors comparing DNA evolution models and the use of a strict and relaxed molecular clock were calculated. Values showing strong evidence for the use of one model are underlined.

We use LRT [57] to evaluate if the HKY85 was a good model of DNA evolution in each of the mammalian species and if the data supported the use of a molecular clock. None of the analyzed data definitively rejected the molecular clock (Table 1), though the *Sus* data p-value was close to significance. The *Mus* and *Sus* data significantly support the use of a more complex model of nucleotide evolution while others are borderline non-significant (*Orcinus* and *Canis*).

When comparing intraspecific models within a Bayesian framework only in two instances did the obtained Bayes factors suggest that one model was more appropriate than another. One was the use of the more complex nucleotide substitution model in the *Homo/Pan* group (although this trend was not observed in the LRT test above). The other one was the support for the use of a relaxed clock in the *Bos/Bison* group. The fact that we are analyzing different species (*Bos taurus*, *Bison bison*, *Bos grunniens*) and the fact that different selective constraints have been pointed out between domesticates and wild individuals of *Bos grunniens* [59] could contribute to the need for a relaxed clock. Another comparison yielded a positive but not strong evidence for the use of one model against another. The two higher values were obtained from comparing the nucleotide substitution model and the strict vs. relaxed clocks in *Mus musculus*. Considering the small generation time and fast mutation rate, it is not surprising that a considerable divergence between different lineages is observed in mouse.

We applied the same correction for purifying selection used previously in the human phylogenetic tree [24] and we compared the ρ age estimates of each node in the trees of the analyzed mammals using both the synonymous mutations (and the synonymous clock obtained through CODEML) and the ρ estimates based on the total number of protein-coding variation and its respective clock after correction using the Gompertz functions described above. Each of the comparisons displayed linear fits with very high correlation coefficients (all higher than 0.97) and a relation value close to 1 in all the species (between 0.91 and 1.09), showing the efficiency of the correction applied. Comparisons between synonymous age estimates and corrected protein-coding estimates are shown in Figure S2. This shows that it is possible to account mathematically for the effect of selection in age estimates and that purifying selection is an evolutionary force with a measurable effect. In the supporting information, we included a calculator for the obtained protein-coding time-dependent molecular clock and synonymous clock for each of the species in Supporting Information S1.

## Discussion

Here we analyzed the effect of purifying selection in the protein-coding genes of seven groups of mammals, employing two previously published methodologies demonstrated in human mtDNA [21], [24], which in many ways are complementary. Considering that purifying selection will tend to eliminate lineages carrying deleterious mutations, we should observe two trends in the protein-coding variation in the tree. First, non-synonymous mutations observed will be on average more deleterious in recent branches when compared with older branches (purifying selection is still acting on these recently formed mutations) [5], [7]. Second, we should see a decreasing proportion of non-synonymous to synonymous mutations (that are approximately neutral) when comparing younger and older branches [6], [24].

In terms of the first trend, the results clearly show the effect of purifying selection in the tree. Mutations with higher MutPred pathogenicity scores are significantly associated with lower ρ values (younger nodes) than are mutations with lower MutPred scores. The results in different species are very similar and some of the differences that do occur are explained by the different time depths of the trees for different species. For example, the higher average ρ values for MutPred scores <7 in *Pan* are due to the older coalescence time of *Pan troglodytes* (Figure 2). Remarkably, considering the differences in time depths of the trees and the biology of the different mammals, the average MutPred pathogenicity scores of observed mutations are very similar (between 0.43 and 0.51) as were the distributions of MutPred scores in the different species (Figures 2 and 3). The canine MutPred probability distribution is the most different, with a higher probability of more deleterious mutations in the distribution of the observed variants. This trend could have been caused by artificial selection during dog domestication or in the selection of the initial lineages to be domesticated. However, in the other analyses the dog data do not stand out as different and the average MutPred pathogenicity score of the distribution of observed variants in the canines is similar to those of the other groups. Bjornerdfeldt et al. [60] suggests that a relaxation of the selective constraints occurred following domestication of the dog. However, this was concluded by comparing the short branches in the domestic dog with some long branches representing wolf samples, so the effect they were detecting is the general increase in non-synonymous mutations in young clades that we are also describing here. A test for relaxation due to domestication would require the analysis of recent wolf clades (not available) along with domesticated dog clades with similar time depths in a similar fashion as was done for the yak [59].

The proportion of synonymous variants in the overall protein-coding diversity decreases when we progressively move from young nodes to more ancient nodes and conversely the proportion of non-synonymous variants decreases. This value will tend to a constant that corresponds to the observed proportion of synonymous mutations in the total number of protein-coding mutations after the effect of purifying selection has become negligible. This value also indicates an empirical ratio of the number of non-synonymous mutations in relation to synonymous mutations that are neutral or nearly neutral. Considering the similarity across species of the MutPred pathogenicity score distributions for observed variants, it is not expected that this proportion of synonymous variants is very different between the species (Figure 4). Three datasets (*Homo*, *Canis* and *Bos*) provided quite reliable Gompertz fits, but for four others (*Mus*, *Pan*, *Orcinus* and *Sus*) the curves are very tentative and should be taken with caution. For these, the parameter/that governs the variance of the agglomerated data points was increased to prevent the already small number of nodes in the respective trees from being reduced too drastically. Excluding *Mus*, the asymptotic values are very similar between species, varying between 0.78–0.84. In the case of *Bos*, *Canis*, *Sus* and *Pan* the values are extremely similar (0.82–0.84).

Another value to take directly from the Gompertz function is the Y-intercept that corresponds to a theoretical initial value of the proportion of synonymous/non-synonymous mutations before purifying selection has acted. The initial proportion is very similar for all species (0.57–0.65). The curve of *Mus* should be considered very tentative, and is presented in order to illustrate something that looked evident from the data. In *Mus*, the proportion of non-synonymous mutations in younger branches was enormous compared with other lineages (more than 80% in the y-intercept). This is worth investigating further with more data but it could be caused by two factors. The first factor is that the mutation rate in *Mus* is much faster than in the other mammals (Table 1), which together with the low generation time might allow us to see a much narrower time window of recent evolution. However, another important point to take into account is that the sequenced lineages are mainly lab strains and the evolutionary constraints in that environment can be extremely relaxed.

We provided two molecular clocks estimated using maximum likelihood (Table 1) that researchers can use when investigating the history of the species in question, a synonymous variation clock and a protein-coding region clock corrected for purifying selection. The high correlation between both estimates show the reliability of the calculated Gompertz functions and how the overall patterns of the effect of purifying selection on nonsynonymous variants can be estimated and mathematically incorporated in evolutionary models.

We also estimated all the mutation rates using BEAST (Table 2) mainly with the aim of calculating Bayes factors [58] and comparing the results obtained using a relaxed clock. Only the *Bos*/*Bison* groups showed strong evidence for needing the use of a relaxed clock with the *Mus musculus* showing positive (but not strong) evidence. Three out of six groups showed weak support for a relaxed clock.

It is difficult to directly compare the mutation rates obtained here with rates from other studies since the mtDNA region we analyzed often did not match those employed in other studies. Welch et al. [61] estimated an average rate for mammalian mtDNA of 1.09×10^−8^ mutations/site/year (adding synonymous and non-synonymous branch lengths and averaging all the pairs in the analysis) which is similar to our estimate of 9.04×10^−9^ mutations/site/year. Pesole et al [62] estimated an average mutation rate of 2.96×10^−8^ mutations/site/year for the protein-coding genes, which is three times faster but still comparable with ours considering the smaller dataset available then.

In our analysis, the *Mus* mutation rate estimate is the most divergent. The other five estimates are comparable (Table 1) with the relative rate of 1.4 between *Bos* and *Homo* representing the most divergent pair. *Homo*/*Pan* yielded the second fastest mutation rate. Using the *Orcinus* as a representative of Cetacea in terms of mutation rates [63] and comparing this value with their *Homo*/*Pan* estimated rate the two rates are similar to that obtained in Nabholz et al [64]. However, in that analysis the relative difference between the *Mus*-*Rattus* rate and these two is extreme (about 25 times faster) when compared with the difference obtained here (about 2–3 times faster) where the third position rate of this pair takes the value of 22.3×10^−8^ mutations/site/year. This value can be roughly compared to our synonymous estimate that takes the value of 7.72×10^−8^ mutations/codon/year (Table 1) or 2.57×10^−8^ synonymous mutation/site/year. Goios et al. [65] estimated a rate for protein-coding genes of 13.2×10^−8^ mutations/site/year which is also faster (about 4.5 times) and Welch et al. [61] presents a slower rate of 1.33×10^−8^ mutations/site/year. The discrepancy between these last two studies is due to the calibration points used, which was 12 My for the *Mus*/*Rattus* split in the first and 29.1 My in the latter. We used 12 My as a prior in the relaxed clock analysis but the calibration point used in the strict clock analysis was the separation obtained between *Mus musculus* and *Mus terricolor* since the *Rattus* clearly failed the strict clock test both when analyzed as a genus alone and when incorporating it with the *Mus* (data not shown). Overall it is very unlikely that mammalian mutation rates are so divergent as suggested by Nabholz and colleagues [64]. Gissi et al [66] compared the mtDNA mutation rates of different mammalian groups and they did not vary more than two-fold between them.

The value of 1.29×10^−8^ mutations/site/year in the protein-coding genes obtained from the human/chimpanzee split is very similar to the one obtained by Mishmar et al. [17] for the coding region of 1.26×10^−8^ mutations/site/year. Both studies used a very similar calibration point (6.98 My in this study against 6.5 My in the latter). mtDNA mutation rates in humans are widely studied and one important issue is the question of whether the strict molecular clock is appropriate or even if a molecular clock exists. Although only using African data and the protein-coding region the tests for both the use of a clock (LRT – Table 1) and comparing a strict against a relaxed clock (Bayes factors – Table 2) indicated that a strict molecular clock seems appropriate in this case. This does not mean that a relaxed clock should not be used since we are mainly analysing the effect of mutation rate variation between human lineages that has been pointed out before as problematic in human phylogenetic studies. The effect of purifying selection is another issue that can be dealt with by using a relaxed clock.

The mutation rate obtained by Achilli et al [67] using a similar methodology, for *Bos* was 2.043×10^−8^ mutations/site/year for the coding region, which is more than twice that obtained by us for protein-coding genes (9.00×10^−9^ mutations/site/year). The main difference is that the split between *Bos taurus* and *Bison bison* was selected as 2 My in the first study and we obtained a split of 5.63 My in the BEAST analysis. Ho et al. [68] calculated a split time using ancient DNA and a Bayesian framework which yielded the value of 0.4 My. The authors point out that calibrations from ancient DNA are not appropriate to estimate deep splits and a value between 2–2.5 and 8.9 My, as obtained here, would fit better the paleontological record.

Calibration points are probably the major issue in interspecific molecular clock estimates and they can explain the discrepancies between many studies [69]. In this study, we obtained calibration points for the different analyzed mammalian species resulting from a multi-point calibration analysis using a relaxed clock. In this way, the results for each species are not biased by the selection of a calibration point that might prove not to be the best. All the calibration points were based on the same principles. One point that could illustrate the advantage of using multiple calibration points as a prior is the value of the *Homo*/*Pan* split. Although our prior was 8.25 My, this value would probably be considered too high by most researchers. The posterior obtained re-estimated this split point, leading to a value just below 7 My which fits better the appearance of *Sahelanthropus tchadensis,* which many believe to be close to the *Homo/Pan* split [70].

Again, it is important to point out that the clock presented here is an interspecific molecular clock for which we are presenting a correction for purifying selection that allows an approximation to use in intraspecific phylogenies and to study the phylogeography of the species in question. This is one of the approaches suggested by Ho and Larson [38] to take into account the effect of purifying selection. Other authors have also proposed corrections for the time-dependence of the mutation rate [71]–[73]. Here we have presented one approach for several mammalian species that was implemented in human mtDNA [24] and is already widely used in the literature. Contrary to other approaches, the shape of the mtDNA mutation rate curve is completely obtained by phylogenetic analysis. It is anchored by the synonymous rate that should approach linearity through time [6], [7], [8], [19]. The intraspecific curve fitting does not rely on multiple calibration points based on archaeology or palaeontology [39], [71] that are in most cases dubious and uncertain. Such correlations can also suffer greatly from the presence of ancestral polymorphisms. The method presented here also does not require a demographic model that adds another level of uncertainty.

## Supporting Information

Figure S1 **Exponential fits in the analyzed mammals of the selection function for the amino acid variants defined by dividing the observed distributions of pathogenicity scores by the distribution of scores for all possible variants.**
(DOC)

Figure S2 **Correlation between synonymous and overall protein coding age estimates in the mtDNA tree of the analyzed mammals.**
(DOC)

Table S1 **Accession Numbers of the mammalian species used for the individual trees.**
(DOC)

Table S2 **Accession numbers of the mammalian species.**
(DOC)

Table S3 **Accession numbers of a subset of human sequences from Behar et al. (2008) phylogenetic tree, used in the analysis for the 13 protein-coding genes.**
(DOC)

Table S4 **Calibration internal points used for the Beast Analysis.** The median value for the age was inferred from the minimal/maximal constraints reported by Benson and Donoghue (2007) based on fossil data.
(DOC)

Table S5 **Accession numbers of the species used in the mammalian BEAST tree.**
(DOC)

Supporting Information S1 **Calculator for the obtained protein-coding time-dependent molecular clock and synonymous clock for each of the species analyzed.**
(XLS)
