# Supplementary material for: Evaluating Purifying Selection in the Mitochondrial DNA of Various Mammalian Species
Source: PLoS One. 2013 Mar 22;8(3):e58993. doi: 10.1371/journal.pone.0058993 (PMC3606437; doi:10.1371/journal.pone.0058993)

Figure S1. Exponential fits in the analyzed mammals of the selection function for the amino acid variants defined by dividing the observed distributions of pathogenicity scores by the distribution of scores for all possible variants.


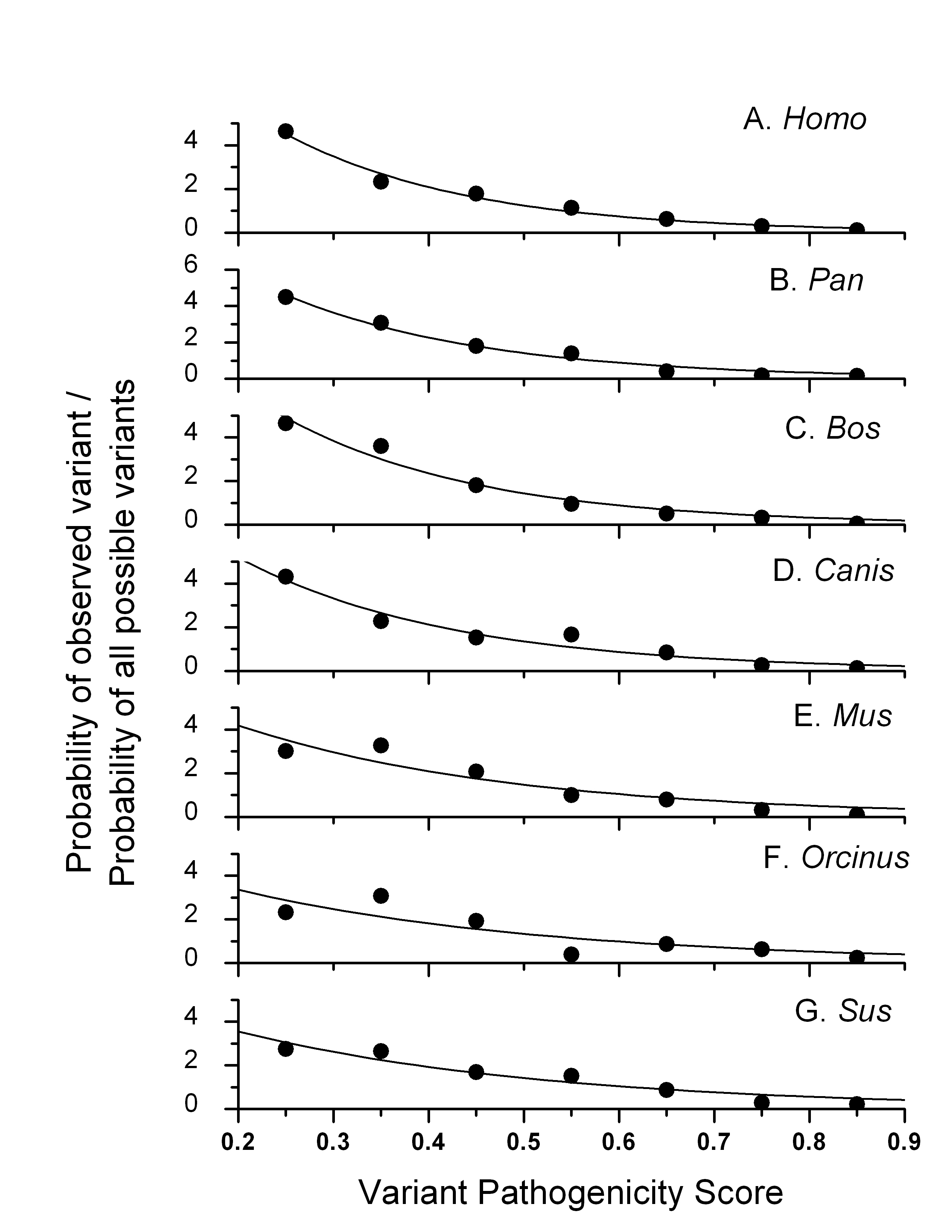

Supplement: Figure S1 — Exponential fits in the analyzed mammals of the selection function for the amino acid variants defined by dividing the observed distributions of pathogenicity scores by the distribution of scores for all possible variants. (DOC) [file pone.0058993.s001.doc]
