# Supplementary material for: Evaluating Purifying Selection in the Mitochondrial DNA of Various Mammalian Species
Source: PLoS One. 2013 Mar 22;8(3):e58993. doi: 10.1371/journal.pone.0058993 (PMC3606437; doi:10.1371/journal.pone.0058993)

Figure S2. Correlation between synonymous and overall protein coding age estimates in the mtDNA tree of the analyzed mammals.


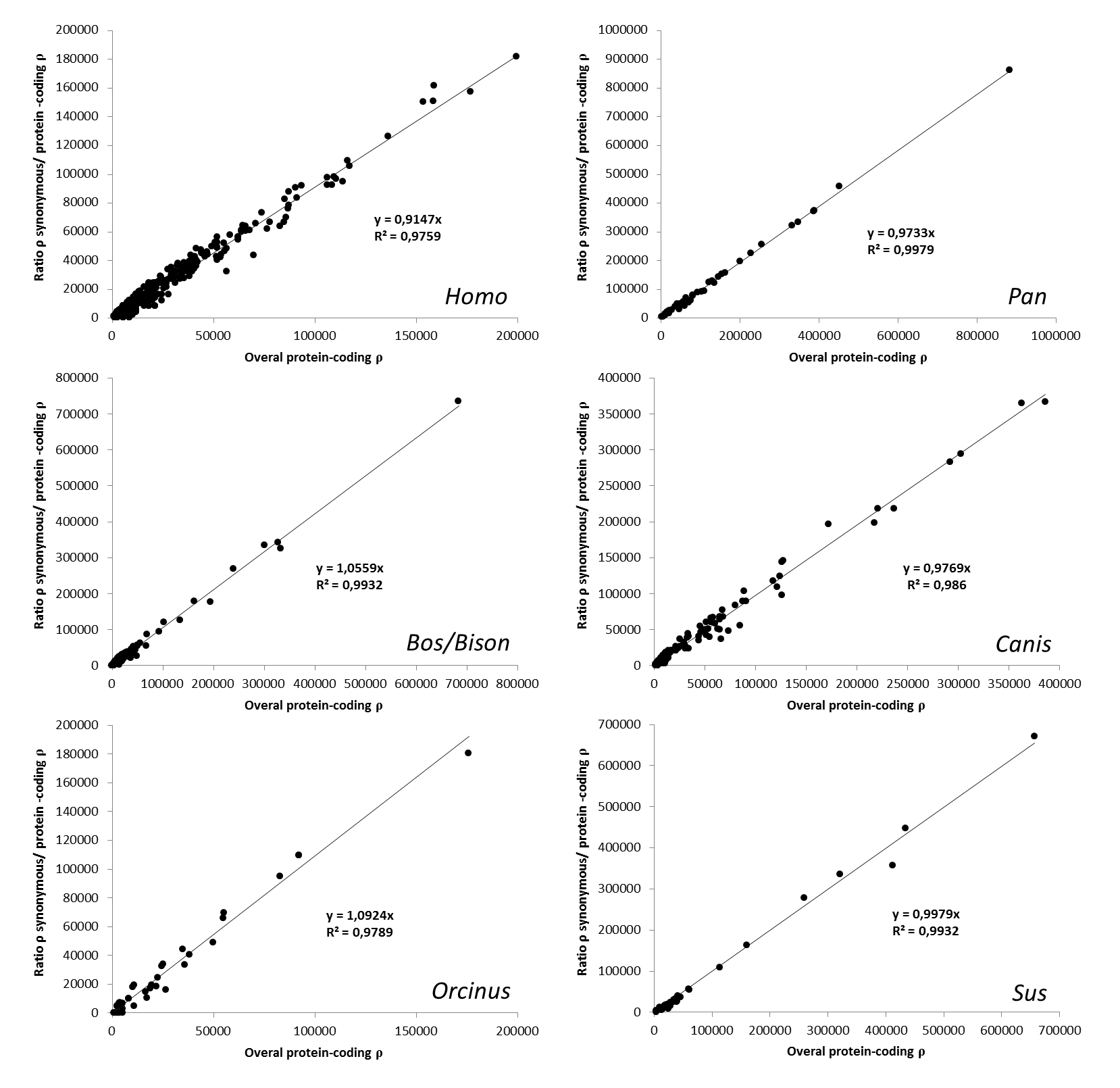

Supplement: Figure S2 — Correlation between synonymous and overall protein coding age estimates in the mtDNA tree of the analyzed mammals. (DOC) [file pone.0058993.s002.doc]
