# Supplementary material for: Evaluating Purifying Selection in the Mitochondrial DNA of Various Mammalian Species
Source: PLoS One. 2013 Mar 22;8(3):e58993. doi: 10.1371/journal.pone.0058993 (PMC3606437; doi:10.1371/journal.pone.0058993)
Supplement: Table S1 — Accession Numbers of the mammalian species used for the individual trees. (DOC) [file pone.0058993.s003.doc]

Table S1 – Accession Numbers of the mammalian species used for the individual trees.

| **Species** | **Accession Number** |
| --- | --- |
| Canis lupus familiaris | AB499817 |
| Canis lupus familiaris | AY656737 |
| Canis lupus familiaris | AY656738 |
| Canis lupus familiaris | AY656739 |
| Canis lupus familiaris | AY656740 |
| Canis lupus familiaris | AY656741 |
| Canis lupus familiaris | AY656742 |
| Canis lupus familiaris | AY656743 |
| Canis lupus familiaris | AY656744 |
| Canis lupus familiaris | AY656745 |
| Canis lupus familiaris | AY656746 |
| Canis lupus familiaris | AY656747 |
| Canis lupus familiaris | AY656748 |
| Canis lupus familiaris | AY656749 |
| Canis lupus familiaris | AY656750 |
| Canis lupus familiaris | AY656751 |
| Canis lupus familiaris | AY656752 |
| Canis lupus familiaris | AY656753 |
| Canis lupus familiaris | AY656754 |
| Canis lupus familiaris | AY656755 |
| Canis lupus familiaris | DQ480489 |
| Canis lupus familiaris | DQ480490 |
| Canis lupus familiaris | DQ480491 |
| Canis lupus familiaris | DQ480492 |
| Canis lupus familiaris | DQ480493 |
| Canis lupus familiaris | DQ480494 |
| Canis lupus familiaris | DQ480495 |
| Canis lupus familiaris | DQ480496 |
| Canis lupus familiaris | DQ480497 |
| Canis lupus familiaris | DQ480498 |
| Canis lupus familiaris | DQ480499 |
| Canis lupus familiaris | DQ480500 |
| Canis lupus familiaris | DQ480501 |
| Canis lupus familiaris | DQ480502 |
| Canis lupus familiaris | DQ480504 |
| Canis lupus familiaris | DQ480505 |
| Canis lupus familiaris | DQ480506 |
| Canis lupus familiaris | DQ480507 |
| Canis lupus familiaris | EU408245 |
| Canis lupus familiaris | EU408246 |
| Canis lupus familiaris | EU408247 |
| Canis lupus familiaris | EU408248 |
| Canis lupus familiaris | EU408249 |
| Canis lupus familiaris | EU408250 |
| Canis lupus familiaris | EU408252 |
| Canis lupus familiaris | EU408253 |
| Canis lupus familiaris | EU408254 |
| Canis lupus familiaris | EU408255 |
| Canis lupus familiaris | EU408256 |
| Canis lupus familiaris | EU408257 |
| Canis lupus familiaris | EU408258 |
| Canis lupus familiaris | EU408259 |
| Canis lupus familiaris | EU408260 |
| Canis lupus familiaris | EU408261 |
| Canis lupus familiaris | EU408262 |
| Canis lupus familiaris | EU408263 |
| Canis lupus familiaris | EU408264 |
| Canis lupus familiaris | EU408265 |
| Canis lupus familiaris | EU408266 |
| Canis lupus familiaris | EU408267 |
| Canis lupus familiaris | EU408268 |
| Canis lupus familiaris | EU408269 |
| Canis lupus familiaris | EU408270 |
| Canis lupus familiaris | EU408271 |
| Canis lupus familiaris | EU408272 |
| Canis lupus familiaris | EU408273 |
| Canis lupus familiaris | EU408274 |
| Canis lupus familiaris | EU408275 |
| Canis lupus familiaris | EU408276 |
| Canis lupus familiaris | EU408277 |
| Canis lupus familiaris | EU408278 |
| Canis lupus familiaris | EU408279 |
| Canis lupus familiaris | EU408280 |
| Canis lupus familiaris | EU408281 |
| Canis lupus familiaris | EU408282 |
| Canis lupus familiaris | EU408283 |
| Canis lupus familiaris | EU408284 |
| Canis lupus familiaris | EU408285 |
| Canis lupus familiaris | EU408286 |
| Canis lupus familiaris | EU408287 |
| Canis lupus familiaris | EU408288 |
| Canis lupus familiaris | EU408289 |
| Canis lupus familiaris | EU408290 |
| Canis lupus familiaris | EU408291 |
| Canis lupus familiaris | EU408292 |
| Canis lupus familiaris | EU408293 |
| Canis lupus familiaris | EU408294 |
| Canis lupus familiaris | EU408295 |
| Canis lupus familiaris | EU408296 |
| Canis lupus familiaris | EU408297 |
| Canis lupus familiaris | EU408298 |
| Canis lupus familiaris | EU408299 |
| Canis lupus familiaris | EU408300 |
| Canis lupus familiaris | EU408301 |
| Canis lupus familiaris | EU408302 |
| Canis lupus familiaris | EU408303 |
| Canis lupus familiaris | EU408304 |
| Canis lupus familiaris | EU408305 |
| Canis lupus familiaris | EU408306 |
| Canis lupus familiaris | EU408307 |
| Canis lupus familiaris | EU408308 |
| Canis lupus familiaris | EU789638 |
| Canis lupus familiaris | EU789639 |
| Canis lupus familiaris | EU789640 |
| Canis lupus familiaris | EU789641 |
| Canis lupus familiaris | EU789642 |
| Canis lupus familiaris | EU789643 |
| Canis lupus familiaris | EU789644 |
| Canis lupus familiaris | EU789645 |
| Canis lupus familiaris | EU789646 |
| Canis lupus familiaris | EU789647 |
| Canis lupus familiaris | EU789648 |
| Canis lupus familiaris | EU789649 |
| Canis lupus familiaris | EU789650 |
| Canis lupus familiaris | EU789651 |
| Canis lupus familiaris | EU789652 |
| Canis lupus familiaris | EU789653 |
| Canis lupus familiaris | EU789654 |
| Canis lupus familiaris | EU789655 |
| Canis lupus familiaris | EU789656 |
| Canis lupus familiaris | EU789657 |
| Canis lupus familiaris | EU789658 |
| Canis lupus familiaris | EU789659 |
| Canis lupus familiaris | EU789660 |
| Canis lupus familiaris | EU789661 |
| Canis lupus familiaris | EU789662 |
| Canis lupus familiaris | EU789663 |
| Canis lupus familiaris | EU789664 |
| Canis lupus familiaris | EU789665 |
| Canis lupus familiaris | EU789666 |
| Canis lupus familiaris | EU789667 |
| Canis lupus familiaris | EU789668 |
| Canis lupus familiaris | EU789669 |
| Canis lupus familiaris | EU789670 |
| Canis lupus familiaris | EU789671 |
| Canis lupus familiaris | EU789673 |
| Canis lupus familiaris | EU789674 |
| Canis lupus familiaris | EU789675 |
| Canis lupus familiaris | EU789676 |
| Canis lupus familiaris | EU789677 |
| Canis lupus familiaris | EU789678 |
| Canis lupus familiaris | EU789679 |
| Canis lupus familiaris | EU789680 |
| Canis lupus familiaris | EU789681 |
| Canis lupus familiaris | EU789682 |
| Canis lupus familiaris | EU789683 |
| Canis lupus familiaris | EU789684 |
| Canis lupus familiaris | EU789685 |
| Canis lupus familiaris | EU789686 |
| Canis lupus familiaris | EU789687 |
| Canis lupus familiaris | EU789688 |
| Canis lupus familiaris | EU789689 |
| Canis lupus familiaris | EU789690 |
| Canis lupus familiaris | EU789691 |
| Canis lupus familiaris | EU789692 |
| Canis lupus familiaris | EU789693 |
| Canis lupus familiaris | EU789694 |
| Canis lupus familiaris | EU789695 |
| Canis lupus familiaris | EU789696 |
| Canis lupus familiaris | EU789697 |
| Canis lupus familiaris | EU789698 |
| Canis lupus familiaris | EU789699 |
| Canis lupus familiaris | EU789700 |
| Canis lupus familiaris | EU789701 |
| Canis lupus familiaris | EU789702 |
| Canis lupus familiaris | EU789703 |
| Canis lupus familiaris | EU789704 |
| Canis lupus familiaris | EU789705 |
| Canis lupus familiaris | EU789706 |
| Canis lupus familiaris | EU789707 |
| Canis lupus familiaris | EU789708 |
| Canis lupus familiaris | EU789709 |
| Canis lupus familiaris | EU789710 |
| Canis lupus familiaris | EU789711 |
| Canis lupus familiaris | EU789712 |
| Canis lupus familiaris | EU789713 |
| Canis lupus familiaris | EU789714 |
| Canis lupus familiaris | EU789715 |
| Canis lupus familiaris | EU789716 |
| Canis lupus familiaris | EU789717 |
| Canis lupus familiaris | EU789718 |
| Canis lupus familiaris | EU789719 |
| Canis lupus familiaris | EU789720 |
| Canis lupus familiaris | EU789721 |
| Canis lupus familiaris | EU789722 |
| Canis lupus familiaris | EU789723 |
| Canis lupus familiaris | EU789724 |
| Canis lupus familiaris | EU789725 |
| Canis lupus familiaris | EU789726 |
| Canis lupus familiaris | EU789727 |
| Canis lupus familiaris | EU789728 |
| Canis lupus familiaris | EU789729 |
| Canis lupus familiaris | EU789730 |
| Canis lupus familiaris | EU789731 |
| Canis lupus familiaris | EU789732 |
| Canis lupus familiaris | EU789733 |
| Canis lupus familiaris | EU789734 |
| Canis lupus familiaris | EU789735 |
| Canis lupus familiaris | EU789736 |
| Canis lupus familiaris | EU789737 |
| Canis lupus familiaris | EU789738 |
| Canis lupus familiaris | EU789739 |
| Canis lupus familiaris | EU789741 |
| Canis lupus familiaris | EU789742 |
| Canis lupus familiaris | EU789743 |
| Canis lupus familiaris | EU789744 |
| Canis lupus familiaris | EU789745 |
| Canis lupus familiaris | EU789746 |
| Canis lupus familiaris | EU789747 |
| Canis lupus familiaris | EU789748 |
| Canis lupus familiaris | EU789749 |
| Canis lupus familiaris | EU789750 |
| Canis lupus familiaris | EU789751 |
| Canis lupus familiaris | EU789752 |
| Canis lupus familiaris | EU789753 |
| Canis lupus familiaris | EU789754 |
| Canis lupus familiaris | EU789755 |
| Canis lupus familiaris | EU789756 |
| Canis lupus familiaris | EU789757 |
| Canis lupus familiaris | EU789758 |
| Canis lupus familiaris | EU789759 |
| Canis lupus familiaris | EU789760 |
| Canis lupus familiaris | EU789761 |
| Canis lupus familiaris | EU789762 |
| Canis lupus familiaris | EU789763 |
| Canis lupus familiaris | EU789764 |
| Canis lupus familiaris | EU789765 |
| Canis lupus familiaris | EU789766 |
| Canis lupus familiaris | EU789767 |
| Canis lupus familiaris | EU789768 |
| Canis lupus familiaris | EU789769 |
| Canis lupus familiaris | EU789770 |
| Canis lupus familiaris | EU789771 |
| Canis lupus familiaris | EU789772 |
| Canis lupus familiaris | EU789773 |
| Canis lupus familiaris | EU789774 |
| Canis lupus familiaris | EU789775 |
| Canis lupus familiaris | EU789776 |
| Canis lupus familiaris | EU789777 |
| Canis lupus familiaris | EU789778 |
| Canis lupus familiaris | EU789779 |
| Canis lupus familiaris | EU789780 |
| Canis lupus familiaris | EU789781 |
| Canis lupus familiaris | EU789782 |
| Canis lupus familiaris | EU789783 |
| Canis lupus familiaris | EU789784 |
| Canis lupus familiaris | EU789785 |
| Canis lupus familiaris | EU789786 |
| Canis lupus familiaris | EU789787 |
| Canis lupus familiaris | EU789788 |
| Canis lupus familiaris | FJ817362 |
| Canis lupus familiaris | FJ817363 |
| Canis lupus familiaris | FJ817364 |
| Canis lupus familiaris | HM048871 |
| Canis lupus familiaris | NC_002008 |
| Canis lupus lupus | AB499818 |
| Canis lupus lupus | AB499819 |
| Canis lupus lupus | AB499820 |
| Canis lupus lupus | AB499823 |
| Canis lupus lupus | AB499824 |
| Canis lupus lupus | AB499825 |
| Canis lupus lupus | NC_009686 |
| Bison bison | GU946976 |
| Bison bison | GU946977 |
| Bison bison | GU946978 |
| Bison bison | GU946979 |
| Bison bison | GU946980 |
| Bison bison | GU946981 |
| Bison bison | GU946982 |
| Bison bison | GU946983 |
| Bison bison | GU946984 |
| Bison bison | GU946985 |
| Bison bison | GU946986 |
| Bison bison | GU946987 |
| Bison bison | GU946988 |
| Bison bison | GU946989 |
| Bison bison | GU946990 |
| Bison bison | GU946991 |
| Bison bison | GU946992 |
| Bison bison | GU946993 |
| Bison bison | GU946994 |
| Bison bison | GU946995 |
| Bison bison | GU946996 |
| Bison bison | GU946997 |
| Bison bison | GU946998 |
| Bison bison | GU946999 |
| Bison bison | GU947000 |
| Bison bison | GU947001 |
| Bison bison | GU947002 |
| Bison bison | GU947003 |
| Bison bison | GU947004 |
| Bison bison | GU947005 |
| Bison bison | GU947006 |
| Bison bison | NC_012346 |
| Bos grunniens | EF494177 |
| Bos grunniens | EF494178 |
| Bos grunniens | GQ464246 |
| Bos grunniens | GQ464247 |
| Bos grunniens | GQ464248 |
| Bos grunniens | GQ464249 |
| Bos grunniens | GQ464250 |
| Bos grunniens | GQ464252 |
| Bos grunniens | GQ464253 |
| Bos grunniens | GQ464254 |
| Bos grunniens | GQ464255 |
| Bos grunniens | GQ464256 |
| Bos grunniens | GQ464257 |
| Bos grunniens | GQ464258 |
| Bos grunniens | GQ464259 |
| Bos grunniens | GQ464260 |
| Bos grunniens | GQ464261 |
| Bos grunniens | GQ464262 |
| Bos grunniens | GQ464263 |
| Bos grunniens | GQ464264 |
| Bos grunniens | GQ464265 |
| Bos grunniens | GQ464266 |
| Bos grunniens | GQ464267 |
| Bos grunniens | GQ464268 |
| Bos grunniens | GQ464269 |
| Bos grunniens | GQ464270 |
| Bos grunniens | GQ464271 |
| Bos grunniens | GQ464272 |
| Bos grunniens | GQ464273 |
| Bos grunniens | GQ464274 |
| Bos grunniens | GQ464275 |
| Bos grunniens | GQ464277 |
| Bos grunniens | GQ464278 |
| Bos grunniens | GQ464279 |
| Bos grunniens | GQ464280 |
| Bos grunniens | GQ464281 |
| Bos grunniens | GQ464282 |
| Bos grunniens | GQ464283 |
| Bos grunniens | GQ464284 |
| Bos grunniens | GQ464285 |
| Bos grunniens | GQ464286 |
| Bos grunniens | GQ464287 |
| Bos grunniens | GQ464288 |
| Bos grunniens | GQ464290 |
| Bos grunniens | GQ464291 |
| Bos grunniens | GQ464292 |
| Bos grunniens | GQ464293 |
| Bos grunniens | GQ464294 |
| Bos grunniens | GQ464295 |
| Bos grunniens | GQ464296 |
| Bos grunniens | GQ464297 |
| Bos grunniens | GQ464298 |
| Bos grunniens | GQ464299 |
| Bos grunniens | GQ464300 |
| Bos grunniens | GQ464301 |
| Bos grunniens | GQ464302 |
| Bos grunniens | GQ464303 |
| Bos grunniens | GQ464304 |
| Bos grunniens | GQ464305 |
| Bos grunniens | GQ464306 |
| Bos grunniens | GQ464307 |
| Bos grunniens | GQ464308 |
| Bos grunniens | GQ464309 |
| Bos grunniens | GQ464310 |
| Bos grunniens | GQ464312 |
| Bos grunniens | GQ464314 |
| Bos grunniens | NC_006380 |
| Bos indicus | AF492350 |
| Bos indicus | GU256940 |
| Bos indicus | NC_005971 |
| Bos javanicus | NC_012706 |
| Bos primigenius | NC_013996 |
| Bos taurus | AB074962 |
| Bos taurus | AB074963 |
| Bos taurus | AB074964 |
| Bos taurus | AB074965 |
| Bos taurus | AB074966 |
| Bos taurus | AB074967 |
| Bos taurus | AB074968 |
| Bos taurus | AF492351 |
| Bos taurus | AY676855 |
| Bos taurus | AY676856 |
| Bos taurus | AY676857 |
| Bos taurus | AY676858 |
| Bos taurus | AY676859 |
| Bos taurus | AY676860 |
| Bos taurus | AY676861 |
| Bos taurus | AY676862 |
| Bos taurus | AY676863 |
| Bos taurus | AY676864 |
| Bos taurus | AY676865 |
| Bos taurus | AY676866 |
| Bos taurus | AY676867 |
| Bos taurus | AY676868 |
| Bos taurus | AY676869 |
| Bos taurus | AY676870 |
| Bos taurus | AY676871 |
| Bos taurus | AY676872 |
| Bos taurus | AY676873 |
| Bos taurus | DQ124371 |
| Bos taurus | DQ124372 |
| Bos taurus | DQ124373 |
| Bos taurus | DQ124374 |
| Bos taurus | DQ124375 |
| Bos taurus | DQ124376 |
| Bos taurus | DQ124377 |
| Bos taurus | DQ124378 |
| Bos taurus | DQ124379 |
| Bos taurus | DQ124380 |
| Bos taurus | DQ124381 |
| Bos taurus | DQ124382 |
| Bos taurus | DQ124383 |
| Bos taurus | DQ124384 |
| Bos taurus | DQ124385 |
| Bos taurus | DQ124386 |
| Bos taurus | DQ124387 |
| Bos taurus | DQ124388 |
| Bos taurus | DQ124389 |
| Bos taurus | DQ124390 |
| Bos taurus | DQ124391 |
| Bos taurus | DQ124392 |
| Bos taurus | DQ124393 |
| Bos taurus | DQ124394 |
| Bos taurus | DQ124395 |
| Bos taurus | DQ124396 |
| Bos taurus | DQ124397 |
| Bos taurus | DQ124398 |
| Bos taurus | DQ124399 |
| Bos taurus | DQ124400 |
| Bos taurus | DQ124401 |
| Bos taurus | DQ124402 |
| Bos taurus | DQ124403 |
| Bos taurus | DQ124404 |
| Bos taurus | DQ124405 |
| Bos taurus | DQ124406 |
| Bos taurus | DQ124407 |
| Bos taurus | DQ124408 |
| Bos taurus | DQ124409 |
| Bos taurus | DQ124410 |
| Bos taurus | DQ124411 |
| Bos taurus | DQ124412 |
| Bos taurus | DQ124413 |
| Bos taurus | DQ124414 |
| Bos taurus | DQ124415 |
| Bos taurus | DQ124416 |
| Bos taurus | DQ124417 |
| Bos taurus | DQ124418 |
| Bos taurus | EU177815 |
| Bos taurus | EU177816 |
| Bos taurus | EU177817 |
| Bos taurus | EU177818 |
| Bos taurus | EU177819 |
| Bos taurus | EU177820 |
| Bos taurus | EU177821 |
| Bos taurus | EU177822 |
| Bos taurus | EU177823 |
| Bos taurus | EU177824 |
| Bos taurus | EU177825 |
| Bos taurus | EU177826 |
| Bos taurus | EU177827 |
| Bos taurus | EU177828 |
| Bos taurus | EU177829 |
| Bos taurus | EU177830 |
| Bos taurus | EU177831 |
| Bos taurus | EU177832 |
| Bos taurus | EU177833 |
| Bos taurus | EU177834 |
| Bos taurus | EU177835 |
| Bos taurus | EU177836 |
| Bos taurus | EU177837 |
| Bos taurus | EU177838 |
| Bos taurus | EU177839 |
| Bos taurus | EU177840 |
| Bos taurus | EU177841 |
| Bos taurus | EU177842 |
| Bos taurus | EU177843 |
| Bos taurus | EU177844 |
| Bos taurus | EU177845 |
| Bos taurus | EU177846 |
| Bos taurus | EU177847 |
| Bos taurus | EU177848 |
| Bos taurus | EU177849 |
| Bos taurus | EU177850 |
| Bos taurus | EU177851 |
| Bos taurus | EU177852 |
| Bos taurus | EU177853 |
| Bos taurus | EU177854 |
| Bos taurus | EU177855 |
| Bos taurus | EU177856 |
| Bos taurus | EU177857 |
| Bos taurus | EU177858 |
| Bos taurus | EU177859 |
| Bos taurus | EU177860 |
| Bos taurus | EU177861 |
| Bos taurus | EU177862 |
| Bos taurus | EU177863 |
| Bos taurus | EU177864 |
| Bos taurus | EU177865 |
| Bos taurus | EU177866 |
| Bos taurus | EU177867 |
| Bos taurus | EU177868 |
| Bos taurus | EU177869 |
| Bos taurus | EU177870 |
| Bos taurus | FJ971080 |
| Bos taurus | FJ971081 |
| Bos taurus | FJ971082 |
| Bos taurus | FJ971083 |
| Bos taurus | FJ971084 |
| Bos taurus | FJ971085 |
| Bos taurus | FJ971086 |
| Bos taurus | FJ971087 |
| Bos taurus | FJ971088 |
| Bos taurus | GQ129207 |
| Bos taurus | GQ129208 |
| Bos taurus | GU947007 |
| Bos taurus | GU947008 |
| Bos taurus | GU947009 |
| Bos taurus | GU947010 |
| Bos taurus | GU947011 |
| Bos taurus | GU947012 |
| Bos taurus | GU947013 |
| Bos taurus | GU947014 |
| Bos taurus | GU947015 |
| Bos taurus | GU947016 |
| Bos taurus | GU947017 |
| Bos taurus | GU947018 |
| Bos taurus | GU947019 |
| Bos taurus | GU947020 |
| Bos taurus | GU947021 |
| Bos taurus | HM045018 |
| Bos taurus | HQ184030 |
| Bos taurus | HQ184031 |
| Bos taurus | HQ184032 |
| Bos taurus | HQ184033 |
| Bos taurus | HQ184034 |
| Bos taurus | HQ184035 |
| Bos taurus | HQ184036 |
| Bos taurus | HQ184037 |
| Bos taurus | HQ184038 |
| Bos taurus | HQ184039 |
| Bos taurus | HQ184040 |
| Bos taurus | HQ184041 |
| Bos taurus | HQ184042 |
| Bos taurus | HQ184043 |
| Bos taurus | HQ184044 |
| Bos taurus | HQ184045 |
| Bos taurus | NC_006853 |
| Bos taurus | V00654 |
| Mus musculus | AB042523 |
| Mus musculus | AB042524 |
| Mus musculus | AB042809 |
| Mus musculus | AB049357 |
| Mus musculus | AJ489607 |
| Mus musculus | AJ512208 |
| Mus musculus | AY339599 |
| Mus musculus | AY466499 |
| Mus musculus | AY533105 |
| Mus musculus | AY533106 |
| Mus musculus | AY533107 |
| Mus musculus | AY533108 |
| Mus musculus | AY999076 |
| Mus musculus | D83491 |
| Mus musculus | DQ106412 |
| Mus musculus | DQ106413 |
| Mus musculus | EF108330 |
| Mus musculus | EF108331 |
| Mus musculus | EF108332 |
| Mus musculus | EF108333 |
| Mus musculus | EF108334 |
| Mus musculus | EF108335 |
| Mus musculus | EF108336 |
| Mus musculus | EF108337 |
| Mus musculus | EF108338 |
| Mus musculus | EF108339 |
| Mus musculus | EF108340 |
| Mus musculus | EF108341 |
| Mus musculus | EF108343 |
| Mus musculus | EF108344 |
| Mus musculus | EF108345 |
| Mus musculus | EU312160 |
| Mus musculus | EU312161 |
| Mus musculus | EU315228 |
| Mus musculus | EU315229 |
| Mus musculus | EU450583 |
| Mus musculus | FJ374639 |
| Mus musculus | FJ374640 |
| Mus musculus | FJ374641 |
| Mus musculus | FJ374642 |
| Mus musculus | FJ374643 |
| Mus musculus | FJ374644 |
| Mus musculus | FJ374645 |
| Mus musculus | FJ374646 |
| Mus musculus | FJ374647 |
| Mus musculus | FJ374648 |
| Mus musculus | FJ374649 |
| Mus musculus | FJ374650 |
| Mus musculus | FJ374651 |
| Mus musculus | FJ374652 |
| Mus musculus | FJ374653 |
| Mus musculus | FJ374654 |
| Mus musculus | FJ374655 |
| Mus musculus | FJ374656 |
| Mus musculus | FJ374657 |
| Mus musculus | FJ374658 |
| Mus musculus | FJ374659 |
| Mus musculus | FJ374660 |
| Mus musculus | FJ374661 |
| Mus musculus | FJ374662 |
| Mus musculus | FJ374663 |
| Mus musculus | FJ374664 |
| Mus musculus | FJ374665 |
| Mus musculus | FJ803909 |
| Mus musculus | GQ871744 |
| Mus musculus | GQ871745 |
| Mus musculus | GQ871746 |
| Mus musculus | HQ586004 |
| Mus musculus | MUSMTCG |
| Mus musculus | MUSMTHYPA |
| Mus musculus | MUSMTHYPB |
| Mus musculus | NC_005089 |
| Mus musculus | NC_006914 |
| Mus musculus | NC_006915 |
| Mus musculus | NC_010339 |
| Mus musculus | NC_012387 |
| Mus musculus | V00711 |
| Orcinus orca | GU187155 |
| Orcinus orca | GU187156 |
| Orcinus orca | GU187157 |
| Orcinus orca | GU187158 |
| Orcinus orca | GU187159 |
| Orcinus orca | GU187160 |
| Orcinus orca | GU187161 |
| Orcinus orca | GU187162 |
| Orcinus orca | GU187163 |
| Orcinus orca | GU187164 |
| Orcinus orca | GU187166 |
| Orcinus orca | GU187167 |
| Orcinus orca | GU187168 |
| Orcinus orca | GU187169 |
| Orcinus orca | GU187170 |
| Orcinus orca | GU187171 |
| Orcinus orca | GU187172 |
| Orcinus orca | GU187173 |
| Orcinus orca | GU187174 |
| Orcinus orca | GU187175 |
| Orcinus orca | GU187176 |
| Orcinus orca | GU187177 |
| Orcinus orca | GU187178 |
| Orcinus orca | GU187179 |
| Orcinus orca | GU187180 |
| Orcinus orca | GU187181 |
| Orcinus orca | GU187182 |
| Orcinus orca | GU187183 |
| Orcinus orca | GU187184 |
| Orcinus orca | GU187185 |
| Orcinus orca | GU187186 |
| Orcinus orca | GU187187 |
| Orcinus orca | GU187188 |
| Orcinus orca | GU187189 |
| Orcinus orca | GU187190 |
| Orcinus orca | GU187191 |
| Orcinus orca | GU187192 |
| Orcinus orca | GU187193 |
| Orcinus orca | GU187194 |
| Orcinus orca | GU187195 |
| Orcinus orca | GU187196 |
| Orcinus orca | GU187197 |
| Orcinus orca | GU187198 |
| Orcinus orca | GU187199 |
| Orcinus orca | GU187200 |
| Orcinus orca | GU187203 |
| Orcinus orca | GU187204 |
| Orcinus orca | GU187205 |
| Orcinus orca | GU187206 |
| Orcinus orca | GU187207 |
| Orcinus orca | GU187208 |
| Orcinus orca | GU187209 |
| Orcinus orca | GU187210 |
| Orcinus orca | GU187211 |
| Orcinus orca | GU187212 |
| Orcinus orca | GU187213 |
| Orcinus orca | GU187214 |
| Orcinus orca | GU187215 |
| Orcinus orca | GU187216 |
| Orcinus orca | GU187217 |
| Orcinus orca | GU187218 |
| Orcinus orca | GU187219 |
| Orcinus orca | NC_014682 |
| Pan paniscus | GU189657 |
| Pan paniscus | GU189658 |
| Pan paniscus | GU189659 |
| Pan paniscus | GU189660 |
| Pan paniscus | GU189661 |
| Pan paniscus | GU189662 |
| Pan paniscus | GU189663 |
| Pan paniscus | GU189664 |
| Pan paniscus | GU189665 |
| Pan paniscus | GU189666 |
| Pan paniscus | GU189667 |
| Pan paniscus | GU189668 |
| Pan paniscus | GU189669 |
| Pan paniscus | GU189670 |
| Pan paniscus | GU189671 |
| Pan paniscus | GU189672 |
| Pan paniscus | GU189673 |
| Pan paniscus | GU189674 |
| Pan paniscus | GU189675 |
| Pan paniscus | GU189676 |
| Pan paniscus | GU189677 |
| Pan paniscus | HM015213 |
| Pan paniscus | JF727231 |
| Pan paniscus | JF727232 |
| Pan paniscus | JF727227 |
| Pan paniscus | JF727224 |
| Pan paniscus | JF727228 |
| Pan paniscus | JF727238 |
| Pan paniscus | JF727230 |
| Pan paniscus | JF727226 |
| Pan paniscus | JF727220 |
| Pan paniscus | JF727236 |
| Pan paniscus | JF727234 |
| Pan paniscus | JF727229 |
| Pan paniscus | JF727223 |
| Pan paniscus | JF727221 |
| Pan paniscus | JF727237 |
| Pan paniscus | JF727235 |
| Pan paniscus | JF727233 |
| Pan paniscus | JF727225 |
| Pan paniscus | JF727222 |
| Pan paniscus | JF727219 |
| Pan troglodytes | GU112738 |
| Pan troglodytes | GU112739 |
| Pan troglodytes | GU112740 |
| Pan troglodytes | GU112741 |
| Pan troglodytes | GU112742 |
| Pan troglodytes | GU112743 |
| Pan troglodytes | GU112744 |
| Pan troglodytes | GU112745 |
| Pan troglodytes | HM068570 |
| Pan troglodytes | HM068571 |
| Pan troglodytes | HM068572 |
| Pan troglodytes | HM068573 |
| Pan troglodytes | HM068574 |
| Pan troglodytes | HM068575 |
| Pan troglodytes | HM068576 |
| Pan troglodytes | HM068577 |
| Pan troglodytes | HM068578 |
| Pan troglodytes | HM068579 |
| Pan troglodytes | HM068580 |
| Pan troglodytes | HM068581 |
| Pan troglodytes | HM068582 |
| Pan troglodytes | HM068583 |
| Pan troglodytes | HM068584 |
| Pan troglodytes | HM068585 |
| Pan troglodytes | HM068586 |
| Pan troglodytes | HM068587 |
| Pan troglodytes | HM068588 |
| Pan troglodytes | HM068589 |
| Pan troglodytes | HM068590 |
| Pan troglodytes | HM068591 |
| Pan troglodytes | HM068592 |
| Pan troglodytes | HM068593 |
| Pan troglodytes | X93335 |
| Pan troglodytes | JF727162 |
| Pan troglodytes | JF727163 |
| Pan troglodytes | JF727164 |
| Pan troglodytes | JF727165 |
| Pan troglodytes | JF727166 |
| Pan troglodytes | JF727167 |
| Pan troglodytes | JF727168 |
| Pan troglodytes | JF727169 |
| Pan troglodytes | JF727170 |
| Pan troglodytes | JF727171 |
| Pan troglodytes | JF727172 |
| Pan troglodytes | JF727173 |
| Pan troglodytes | JF727174 |
| Pan troglodytes | JF727175 |
| Pan troglodytes | JF727176 |
| Pan troglodytes | JF727177 |
| Pan troglodytes | JF727178 |
| Pan troglodytes | JF727179 |
| Pan troglodytes | JF727180 |
| Pan troglodytes | JF727181 |
| Pan troglodytes | JF727182 |
| Pan troglodytes | JF727183 |
| Pan troglodytes | JF727184 |
| Pan troglodytes | JF727185 |
| Pan troglodytes | JF727186 |
| Pan troglodytes | JF727187 |
| Pan troglodytes | JF727188 |
| Pan troglodytes | JF727189 |
| Pan troglodytes | JF727190 |
| Pan troglodytes | JF727191 |
| Pan troglodytes | JF727192 |
| Pan troglodytes | JF727193 |
| Pan troglodytes | JF727194 |
| Pan troglodytes | JF727195 |
| Pan troglodytes | JF727196 |
| Pan troglodytes | JF727197 |
| Pan troglodytes | JF727198 |
| Pan troglodytes | JF727199 |
| Pan troglodytes | JF727200 |
| Pan troglodytes | JF727201 |
| Pan troglodytes | JF727202 |
| Pan troglodytes | JF727203 |
| Pan troglodytes | JF727204 |
| Pan troglodytes | JF727205 |
| Pan troglodytes | JF727206 |
| Pan troglodytes | JF727207 |
| Pan troglodytes | JF727208 |
| Pan troglodytes | JF727209 |
| Pan troglodytes | JF727210 |
| Pan troglodytes | JF727211 |
| Pan troglodytes | JF727212 |
| Pan troglodytes | JF727213 |
| Pan troglodytes | JF727214 |
| Pan troglodytes | JF727215 |
| Pan troglodytes | JF727216 |
| Pan troglodytes | JF727217 |
| Pan troglodytes | JF727218 |
| Sus scrofa | AB298688 |
| Sus scrofa | AF304201 |
| Sus scrofa | AF304203 |
| Sus scrofa | AF486855 |
| Sus scrofa | AF486856 |
| Sus scrofa | AF486857 |
| Sus scrofa | AF486858 |
| Sus scrofa | AF486859 |
| Sus scrofa | AF486860 |
| Sus scrofa | AF486861 |
| Sus scrofa | AF486862 |
| Sus scrofa | AF486863 |
| Sus scrofa | AF486864 |
| Sus scrofa | AF486865 |
| Sus scrofa | AF486866 |
| Sus scrofa | AF486867 |
| Sus scrofa | AF486868 |
| Sus scrofa | AF486869 |
| Sus scrofa | AF486870 |
| Sus scrofa | AF486871 |
| Sus scrofa | AF486872 |
| Sus scrofa | AF486873 |
| Sus scrofa | AF486874 |
| Sus scrofa | AJ002189 |
| Sus scrofa | DQ972936 |
| Sus scrofa | EF375877 |
| Sus scrofa | EF545567 |
| Sus scrofa | EF545568 |
| Sus scrofa | EF545569 |
| Sus scrofa | EF545570 |
| Sus scrofa | EF545571 |
| Sus scrofa | EF545572 |
| Sus scrofa | EF545573 |
| Sus scrofa | EF545574 |
| Sus scrofa | EF545575 |
| Sus scrofa | EF545576 |
| Sus scrofa | EF545577 |
| Sus scrofa | EF545579 |
| Sus scrofa | EF545580 |
| Sus scrofa | EF545581 |
| Sus scrofa | EF545582 |
| Sus scrofa | EF545583 |
| Sus scrofa | EF545584 |
| Sus scrofa | EF545585 |
| Sus scrofa | EF545586 |
| Sus scrofa | EF545587 |
| Sus scrofa | EF545588 |
| Sus scrofa | EF545589 |
| Sus scrofa | EF545590 |
| Sus scrofa | EF545593 |
| Sus scrofa | EU117375 |
| Sus scrofa | FJ236991 |
| Sus scrofa | FJ236992 |
| Sus scrofa | FJ236993 |
| Sus scrofa | FJ236994 |
| Sus scrofa | FJ236995 |
| Sus scrofa | FJ236996 |
| Sus scrofa | FJ236997 |
| Sus scrofa | FJ236998 |
| Sus scrofa | FJ236999 |
| Sus scrofa | FJ237000 |
| Sus scrofa | FJ237001 |
| Sus scrofa | FJ237002 |
| Sus scrofa | FJ237003 |
| Sus scrofa | GQ220328 |
| Sus scrofa | GQ220329 |
| Sus scrofa | GU147934 |
| Sus scrofa | NC_012095 |
| Sus scrofa | NC_014692 |
