# Supplementary material for: Evaluating Purifying Selection in the Mitochondrial DNA of Various Mammalian Species
Source: PLoS One. 2013 Mar 22;8(3):e58993. doi: 10.1371/journal.pone.0058993 (PMC3606437; doi:10.1371/journal.pone.0058993)
Supplement: Table S2 — Accession numbers of the mammalian species. (DOC) [file pone.0058993.s004.doc]

Table S2- Accession numbers of the mammalian species.

| Acession Number | Species | Acession Number | Species | Acession Number | Species |
| --- | --- | --- | --- | --- | --- |
| NC_004920 | Chrysochloris asiatica | NC_008092 | Canis lupus | NC_009510 | Ammotragus lervia |
| NC_010304 | Eremitalpa granti | NC_008093 | Canis latrans | NC_013069 | Budorcas taxicolor |
| NC_010301 | Dendrohyrax dorsalis | NC_008434 | Vulpes vulpes | NC_005044 | Capra hircus |
| NC_004919 | Procavia capensis | NC_009686 | Canis lupus lupus | NC_012096 | Capricornis crispus |
| NC_004921 | Elephantulus sp. VB001 | NC_011218 | Canis lupus laniger | NC_010640 | Naemorhedus swinhoei |
| NC_004026 | Macroscelides proboscideus | NC_013445 | Cuon alpinus | NC_013751 | Naemorhedus caudatus |
| NC_005129 | Elephas maximus | NC_013700 | Nyctereutes procyonoides | NC_001941 | Ovis aries |
| NC_000934 | Loxodonta africana | NC_010497 | Spilogale putorius | NC_006973 | Cervus nippon yesoensis |
| NC_009574 | Mammut americanum | NC_009692 | Enhydra lutris | NC_006993 | Cervus nippon centralis |
| NC_007596 | Mammuthus primigenius | NC_011358 | Lutra lutra | NC_007704 | Cervus elaphus |
| NC_003314 | Dugong dugon | NC_009685 | Gulo gulo | NC_008462 | Cervus nippon taiouanus |
| NC_010302 | Trichechus manatus | NC_009678 | Martes melampus | NC_013834 | Cervus nippon hortulorum |
| NC_002631 | Echinops telfairi | NC_011579 | Martes zibellina | NC_013836 | Cervus elaphus xanthopygus |
| NC_002078 | Orycteropus afer | NC_012141 | Martes flavigula | NC_013840 | Cervus elaphus yarkandensis |
| NC_004031 | Galeopterus variegatus | NC_009677 | Meles anakuma | NC_014701 | Cervus eldi |
| NC_004028 | Lepus europaeus | NC_011125 | Meles meles | NC_014703 | Cervus elaphus songaricus |
| NC_001913 | Oryctolagus cuniculus | NC_004029 | Odobenus rosmarus rosmarus | NC_008414 | Rusa unicolor swinhoei |
| NC_003033 | Ochotona collaris | NC_004023 | Arctocephalus forsteri | NC_011821 | Hydropotes inermis |
| NC_005358 | Ochotona princeps | NC_008417 | Arctocephalus pusillus | NC_008749 | Elaphodus cephalophus |
| NC_011029 | Ochotona curzoniae | NC_008420 | Arctocephalus townsendi | NC_004069 | Muntiacus reevesi |
| NC_015112 | Heterocephalus glaber | NC_008415 | Callorhinus ursinus | NC_004563 | Muntiacus muntjak |
| NC_000884 | Cavia porcellus | NC_004030 | Eumetopias jubatus | NC_004577 | Muntiacus crinifrons |
| NC_002658 | Thryonomys swinderianus | NC_008419 | Neophoca cinerea | NC_008491 | Muntiacus reevesi micrurus |
| NC_009056 | Anomalurus sp. GP-2005 | NC_008418 | Phocarctos hookeri | NC_007703 | Rangifer tarandus |
| NC_005314 | Jaculus jaculus | NC_008416 | Zalophus californianus | NC_012100 | Giraffa camelopardalis angolensis |
| NC_001892 | Glis glis | NC_008427 | Cystophora cristata | NC_012694 | Moschus berezovskii |
| NC_013571 | Eothenomys chinensis | NC_008426 | Erignathus barbatus | NC_013753 | Moschus moschiferus |
| NC_003041 | Microtus kikuchii | NC_001602 | Halichoerus grypus | NC_008830 | Phacochoerus africanus |
| NC_008064 | Microtus levis | NC_008425 | Hydrurga leptonyx | NC_000845 | Sus scrofa |
| NC_015241 | Microtus fortis fortis | NC_008424 | Leptonychotes weddellii | NC_012095 | Sus scrofa domesticus |
| NC_015243 | Microtus fortis calamorum | NC_008423 | Lobodon carcinophaga | NC_014692 | Sus scrofa taiwanensis |
| NC_013563 | Proedromys liangshanensis | NC_008422 | Mirounga leonina | NC_012103 | Pecari tajacu |
| NC_007936 | Cricetulus griseus | NC_008421 | Monachus schauinslandi | NC_009628 | Camelus bactrianus |
| NC_013276 | Mesocricetus auratus | NC_001325 | Phoca vitulina | NC_009629 | Camelus ferus |
| NC_013068 | Tscherskia triton | NC_008428 | Phoca fasciata | NC_009849 | Camelus dromedarius |
| NC_014696 | Leggadina lakedownensis | NC_008429 | Phoca groenlandica | NC_002504 | Lama pacos |
| NC_005089 | Mus musculus | NC_008430 | Phoca largha | NC_011822 | Lama guanicoe |
| NC_006914 | Mus musculus domesticus | NC_008431 | Pusa caspica | NC_012102 | Lama glama |
| NC_006915 | Mus musculus molossinus | NC_008432 | Pusa sibirica | NC_013558 | Vicugna vicugna |
| NC_010339 | Mus musculus musculus | NC_008433 | Pusa hispida | NC_002612 | Pteropus dasymallus |
| NC_010650 | Mus terricolor | NC_009126 | Procyon lotor | NC_002619 | Pteropus scapulatus |
| NC_012387 | Mus musculus castaneus | NC_009492 | Ailuropoda melanoleuca | NC_007393 | Rousettus aegyptiacus |
| NC_014698 | Pseudomys chapmani | NC_011116 | Arctodus simus | NC_006925 | Mystacina tuberculata |
| NC_001665 | Rattus norvegicus | NC_009968 | Helarctos malayanus | NC_002009 | Artibeus jamaicensis |
| NC_011638 | Rattus tanezumi | NC_009970 | Melursus ursinus | NC_005433 | Rhinolophus monoceros |
| NC_012374 | Rattus rattus | NC_009969 | Tremarctos ornatus | NC_005434 | Rhinolophus pumilus |
| NC_012389 | Rattus exulans | NC_003426 | Ursus americanus | NC_011304 | Rhinolophus formosae |
| NC_012461 | Rattus praetor | NC_003427 | Ursus arctos | NC_002626 | Chalinolobus tuberculatus |
| NC_014855 | Rattus leucopus | NC_003428 | Ursus maritimus | NC_005436 | Pipistrellus abramus |
| NC_014858 | Rattus lutreolus | NC_008753 | Ursus thibetanus mupinensis | NC_002080 | Erinaceus europaeus |
| NC_014861 | Rattus tunneyi | NC_009331 | Ursus thibetanus formosanus | NC_005033 | Hemiechinus auritus |
| NC_014864 | Rattus villosissimus | NC_009971 | Ursus thibetanus | NC_002808 | Echinosorex gymnura |
| NC_014867 | Rattus fuscipes | NC_011112 | Ursus spelaeus | NC_010298 | Hylomys suillus |
| NC_014871 | Rattus sordidus | NC_011117 | Ursus thibetanus ussuricus | NC_006893 | Crocidura russula |
| NC_005315 | Nannospalax ehrenbergi | NC_011118 | Ursus thibetanus thibetanus | NC_003040 | Episoriculus fumidus |
| NC_002369 | Sciurus vulgaris | NC_005212 | Acinonyx jubatus | NC_005435 | Sorex unguiculatus |
| NC_009747 | Chlorocebus pygerythrus | NC_001700 | Felis catus | NC_008156 | Galemys pyrenaicus |
| NC_009748 | Chlorocebus tantalus | NC_014456 | Lynx rufus | NC_005035 | Mogera wogura |
| NC_002764 | Macaca sylvanus | NC_008450 | Neofelis nebulosa | NC_002391 | Talpa europaea |
| NC_005943 | Macaca mulatta | NC_010641 | Panthera pardus | NC_005034 | Urotrichus talpoides |
| NC_011519 | Macaca thibetana | NC_010642 | Panthera tigris | NC_001640 | Equus caballus |
| NC_012670 | Macaca fascicularis | NC_014770 | Panthera tigris amoyensis | NC_001788 | Equus asinus |
| NC_001992 | Papio hamadryas | NC_010638 | Uncia uncia | NC_012681 | Coelodonta antiquitatis |
| NC_006901 | Colobus guereza | NC_005268 | Balaena mysticetus | NC_012684 | Dicerorhinus sumatrensis |
| NC_008216 | Nasalis larvatus | NC_006930 | Eubalaena australis | NC_012682 | Diceros bicornis |
| NC_008219 | Piliocolobus badius | NC_001321 | Balaenoptera physalus | NC_001779 | Rhinoceros unicornis |
| NC_008217 | Presbytis melalophos | NC_001601 | Balaenoptera musculus | NC_001808 | Ceratotherium simum |
| NC_008220 | Pygathrix nemaeus | NC_005271 | Balaenoptera acutorostrata | NC_012683 | Rhinoceros sondaicus |
| NC_008218 | Rhinopithecus roxellana | NC_006926 | Balaenoptera bonaerensis | NC_004027 | Manis tetradactyla |
| NC_008215 | Semnopithecus entellus | NC_006928 | Balaenoptera brydei | NC_001821 | Dasypus novemcinctus |
| NC_006900 | Trachypithecus obscurus | NC_006929 | Balaenoptera borealis | NC_006923 | Bradypus tridactylus |
| NC_001645 | Gorilla gorilla | NC_007937 | Balaenoptera omurai | NC_006924 | Choloepus didactylus |
| NC_011120 | Gorilla gorilla gorilla | NC_007938 | Balaenoptera edeni | NC_004032 | Tamandua tetradactyla |
| NC_011137 | Homo sapiens neanderthalensis | NC_006927 | Megaptera novaeangliae | NC_007630 | Dasyurus hallucatus |
| NC_012920 | Homo sapiens | NC_005270 | Eschrichtius robustus | NC_006523 | Phascogale tapoatafa |
| NC_013993 | Homo sp. Altai | NC_005269 | Caperea marginata | NC_006517 | Sminthopsis douglasi |
| NC_001643 | Pan troglodytes | NC_012061 | Delphinus capensis | NC_007631 | Sminthopsis crassicaudata |
| NC_001644 | Pan paniscus | NC_012062 | Grampus griseus | NC_011949 | Myrmecobius fasciatus |
| NC_001646 | Pongo pygmaeus | NC_005278 | Lagenorhynchus albirostris | NC_011944 | Thylacinus cynocephalus |
| NC_002083 | Pongo abelii | NC_014682 | Orcinus orca | NC_001610 | Didelphis virginiana |
| NC_002082 | Hylobates lar | NC_012057 | Sousa chinensis | NC_006516 | Metachirus nudicaudatus |
| NC_014042 | Hylobates agilis | NC_012051 | Stenella attenuata | NC_006299 | Monodelphis domestica |
| NC_014045 | Hylobates pileatus | NC_012053 | Stenella coeruleoalba | NC_005825 | Thylamys elegans |
| NC_014051 | Nomascus siki | NC_012058 | Tursiops aduncus | NC_008145 | Distoechurus pennatus |
| NC_014047 | Symphalangus syndactylus | NC_012059 | Tursiops truncatus | NC_008136 | Lagorchestes hirsutus |
| NC_002763 | Cebus albifrons | NC_005276 | Inia geoffrensis | NC_008447 | Lagostrophus fasciatus |
| NC_012775 | Saimiri sciureus | NC_007629 | Lipotes vexillifer | NC_001794 | Macropus robustus |
| NC_002811 | Tarsius bancanus | NC_005279 | Monodon monoceros | NC_008134 | Dactylopsila trivirgata |
| NC_012774 | Tarsius syrichta | NC_005280 | Phocoena phocoena | NC_008135 | Petaurus breviceps |
| NC_010299 | Daubentonia madagascariensis | NC_005272 | Kogia breviceps | NC_008137 | Phalanger vestitus |
| NC_012763 | Loris tardigradus | NC_002503 | Physeter catodon | NC_003039 | Trichosurus vulpecula |
| NC_002765 | Nycticebus coucang | NC_005275 | Platanista minor | NC_008133 | Phascolarctos cinereus |
| NC_012764 | Perodicticus potto | NC_005277 | Pontoporia blainvillei | NC_006524 | Potorous tridactylus |
| NC_011053 | Propithecus coquereli | NC_005274 | Berardius bairdii | NC_006519 | Pseudocheirus peregrinus |
| NC_010300 | Eulemur mongoz | NC_005273 | Hyperoodon ampullatus | NC_006518 | Tarsipes rostratus |
| NC_012766 | Eulemur fulvus fulvus | NC_000889 | Hippopotamus amphibius | NC_003322 | Vombatus ursinus |
| NC_012769 | Eulemur fulvus mayottensis | NC_012098 | Antilope cervicapra | NC_005826 | Dromiciops gliroides |
| NC_012771 | Eulemur macaco macaco | NC_007441 | Pantholops hodgsonii | NC_006522 | Notoryctes typhlops |
| NC_004025 | Lemur catta | NC_014875 | Procapra przewalskii | NC_005828 | Caenolestes fuliginosus |
| NC_012773 | Varecia variegata variegata | NC_012346 | Bison bison | NC_005829 | Rhyncholestes raphanurus |
| NC_014453 | Lepilemur hubbardorum | NC_014044 | Bison bonasus | NC_002746 | Isoodon macrourus |
| NC_012761 | Galago senegalensis | NC_005971 | Bos indicus | NC_006520 | Macrotis lagotis |
| NC_012762 | Otolemur crassicaudatus | NC_006380 | Bos grunniens | NC_006521 | Perameles gunnii |
| NC_002521 | Tupaia belangeri | NC_006853 | Bos taurus | NC_000891 | Ornithorhynchus anatinus |
| NC_009691 | Ailurus fulgens styani | NC_012706 | Bos javanicus | NC_003321 | Tachyglossus aculeatus |
| NC_011124 | Ailurus fulgens | NC_013996 | Bos primigenius | NC_006364 | Zaglossus bruijni |
| NC_002008 | Canis lupus familiaris | NC_006295 | Bubalus bubalis |  |  |
