# Supplementary material for: Evaluating Purifying Selection in the Mitochondrial DNA of Various Mammalian Species
Source: PLoS One. 2013 Mar 22;8(3):e58993. doi: 10.1371/journal.pone.0058993 (PMC3606437; doi:10.1371/journal.pone.0058993)
Supplement: Table S3 — Accession numbers of a subset of human sequences from Behar et al. (2008) phylogenetic tree, used in the analysis for the 13 protein-coding genes. (DOC) [file pone.0058993.s005.doc]

Table S3 – Accession numbers of a subset of human sequences from Behar et al. (2008) phylogenetic tree, used in the analysis for the 13 protein-coding genes.

| Sample | Accession Number | Sample | Accession Number | Sample | Accession Number |
| --- | --- | --- | --- | --- | --- |
| L506 | EU092966 | L004 | AF346976 | L486 | EU092830 |
| L490 | EU092833 | L471 | EU092816 | L037 | AY195782 |
| L500 | EU092841 | L148P | DQ112698 | L199P | DQ112756 |
| L487 | EU092831 | L345 | EU092711 | L027 | AF381998 |
| L498 | EU092839 | L339 | EU092705 | L528 | EU092867 |
| L499 | EU092840 | L202P | DQ112759 | L309 | EU092675 |
| L342 | EU092708 | L187P | DQ112737 | L375 | EU092740 |
| L583 | EU092921 | L554 | EU092893 | L327 | EU092693 |
| L302 | EU092668 | L605 | EU092940 | L006 | AF346980 |
| L601 | EU092936 | L347 | EU092713 | L364 | EU092729 |
| L474 | EU092819 | L038 | AY195783 | L421 | EU092777 |
| L381 | EU092746 | L590 | EU092928 | L311 | EU092677 |
| L572 | EU092911 | L268 | EU273478 | L329 | EU092695 |
| L015 | AF346998 | L269 | EU273479 | L621 | DQ341070 |
| L561 | EU092900 | L145p | DQ112695 | L585 | EU092923 |
| L567 | EU092906 | L351 | EU092717 | L318 | EU092684 |
| L635 | DQ341060 | L494 | EU092836 | L480 | EU092824 |
| L418 | EU092774 | L280 | EU273489 | L552 | EU092891 |
| L417 | EU092773 | L206p | DQ112794 | L330 | EU092696 |
| L320 | EU092686 | L010 | AF346992 | L398 | EU092758 |
| L359 | EU092724 | L373 | EU092738 | L544 | EU092883 |
| L472 | EU092817 | L337 | EU092703 | L573 | EU092912 |
| L298 | EU092664 | L466 | EU092811 | L414 | EU092770 |
| L326 | EU092692 | L284 | EU273493 | L555 | EU092894 |
| L369 | EU092734 | L384 | EU092748 | L633 | DQ341081 |
| L012 | AF346995 | L463 | EU092808 | L262 | EF556166 |
| L331 | EU092697 | L428 | EU092780 | L484 | EU092828 |
| L543 | EU092882 | L207p | DQ112796 | L632 | DQ341080 |
| L041 | AY195788 | L328 | EU092694 | L293 | EU092660 |
| L442 | EU092793 | L316 | EU092682 | L256p | DQ112956 |
| L354 | EU092720 | L360 | EU092725 |  |  |
| L409 | EU092765 | L569 | EU092908 |  |  |
| L310 | EU092676 | L623 | DQ341072 |  |  |
