# Supplementary material for: Evaluating Purifying Selection in the Mitochondrial DNA of Various Mammalian Species
Source: PLoS One. 2013 Mar 22;8(3):e58993. doi: 10.1371/journal.pone.0058993 (PMC3606437; doi:10.1371/journal.pone.0058993)
Supplement: Table S4 — Calibration internal points used for the Beast Analysis. The median value for the age was inferred from the minimal/maximal constraints reported by Benson and Donoghue (2007) based on fossil data. (DOC) [file pone.0058993.s006.doc]

Table S4 – Calibration internal points used for the Beast Analysis. The median value for the age was inferred from the minimal/maximal constraints reported by Benson and Donoghue (2007) based on fossil data.

| Split/Group | Age median value (MY) | Age minimal/maximal constraints (MY) | SD |
| --- | --- | --- | --- |
| Homo/Pan | 8.25 | 6.5-10 | 0.893 |
| Catarrhini | 28.75 | 23.5-34 | 2.68 |
| Muridae | 11.65 | 11-12.3 | 0.33 |
| Carnivora | 53.4 | 43-63.8 | 5.305 |
| Carnivora/Perissodactyla | 66.75 | 62.3-71.2 | 2.27 |
| Bovidae | 23.425 | 18.3-28.55 | 2.615 |
| Cetartyodactyla | 50.75 | 48-53.5 | 1.405 |
| Cetartyodactyla/(Carnivora/Perissodactyla) | 66.7 | 62.2-71.2 | 2.295 |
| Chiroptera/(Cetartyodactyla/(Carnivora/Perissodactyla)) | 66.35 | 61.5-71.2 | 2.475 |
| Afrotheria | 80.7 | 48.4-113 | 16.48 |
| Marsupials | 66.35 | 61.5-71.2 | 2.475 |
| Mammals | 176.8 | 162.5-191.1 | 7.3 |
