# Supplementary material for: Evaluating Purifying Selection in the Mitochondrial DNA of Various Mammalian Species
Source: PLoS One. 2013 Mar 22;8(3):e58993. doi: 10.1371/journal.pone.0058993 (PMC3606437; doi:10.1371/journal.pone.0058993)
Supplement: Table S5 — Accession numbers of the species used in the mammalian BEAST tree. (DOC) [file pone.0058993.s007.doc]

Table S5. Accession numbers of the species used in the mammalian BEAST tree

| Accession Number | Species |
| --- | --- |
| NC_009492 | Ailuropoda melanoleuca |
| NC_011124 | Ailurus fulgens |
| NC_009056 | Anomalurus sp. GP-2005 |
| NC_012098 | Antilope cervicapra |
| NC_004023 | Arctocephalus forsteri |
| NC_011116 | Arctodus simus |
| NC_002009 | Artibeus jamaicensis |
| NC_005268 | Balaena mysticetus |
| NC_006926 | Balaenoptera bonaerensis |
| NC_001321 | Balaenoptera physalus |
| NC_005274 | Berardius bairdii |
| NC_012346 | Bison bison |
| NC_014044 | Bison bonasus |
| NC_006853 | Bos taurus |
| NC_006923 | Bradypus tridactylus |
| NC_006295 | Bubalus bubalis |
| NC_005828 | Caenolestes fuliginosus |
| NC_009628 | Camelus bactrianus |
| NC_008093 | Canis latrans |
| NC_002008 | Canis lupus familiaris |
| NC_011218 | Canis lupus laniger |
| NC_005044 | Capra hircus |
| NC_000884 | Cavia porcellus |
| NC_002763 | Cebus albifrons |
| NC_001808 | Ceratotherium simum |
| NC_013834 | Cervus nippon hortulorum |
| NC_002626 | Chalinolobus tuberculatus |
| NC_009747 | Chlorocebus pygerythrus |
| NC_009748 | Chlorocebus tantalus |
| NC_004920 | Chrysochloris asiatica |
| NC_006901 | Colobus guereza |
| NC_007936 | Cricetulus griseus |
| NC_006893 | Crocidura russula |
| NC_013445 | Cuon alpinus |
| NC_008134 | Dactylopsila trivirgata |
| NC_001821 | Dasypus novemcinctus |
| NC_007630 | Dasyurus hallucatus |
| NC_010299 | Daubentonia madagascariensis |
| NC_010301 | Dendrohyrax dorsalis |
| NC_012684 | Dicerorhinus sumatrensis |
| NC_001610 | Didelphis virginiana |
| NC_008145 | Distoechurus pennatus |
| NC_005826 | Dromiciops gliroides |
| NC_003314 | Dugong dugon |
| NC_002631 | Echinops telfairi |
| NC_002808 | Echinosorex gymnura |
| NC_008749 | Elaphodus cephalophus |
| NC_005129 | Elephas maximus |
| NC_009692 | Enhydra lutris |
| NC_013571 | Eothenomys chinensis |
| NC_003040 | Episoriculus fumidus |
| NC_001788 | Equus asinus |
| NC_001640 | Equus caballus |
| NC_002080 | Erinaceus europaeus |
| NC_010300 | Eulemur mongoz |
| NC_001700 | Felis catus |
| NC_012761 | Galago senegalensis |
| NC_008156 | Galemys pyrenaicus |
| NC_004031 | Galeopterus variegatus |
| NC_012100 | Giraffa camelopardalis angolensis |
| NC_001892 | Glis glis |
| NC_001645 | Gorilla gorilla |
| NC_012062 | Grampus griseus |
| NC_009685 | Gulo gulo |
| NC_015112 | Heterocephalus glaber |
| NC_000889 | Hippopotamus amphibius |
| NC_012920 | Homo sapiens |
| NC_011821 | Hydropotes inermis |
| NC_008425 | Hydrurga leptonyx |
| NC_014042 | Hylobates agilis |
| NC_005276 | Inia geoffrensis |
| NC_005314 | Jaculus jaculus |
| NC_005272 | Kogia breviceps |
| NC_005278 | Lagenorhynchus albirostris |
| NC_008447 | Lagostrophus fasciatus |
| NC_002504 | Lama pacos/Vicugna pacos |
| NC_014696 | Leggadina lakedownensis |
| NC_004025 | Lemur catta |
| NC_014453 | Lepilemur hubbardorum |
| NC_004028 | Lepus europaeus |
| NC_007629 | Lipotes vexillifer |
| NC_012763 | Loris tardigradus |
| NC_000934 | Loxodonta africana |
| NC_014456 | Lynx rufus |
| NC_012670 | Macaca fascicularis |
| NC_001794 | Macropus robustus |
| NC_004026 | Macroscelides proboscideus |
| NC_006520 | Macrotis lagotis |
| NC_004027 | Manis tetradactyla |
| NC_011579 | Martes zibellina |
| NC_006927 | Megaptera novaeangliae |
| NC_009677 | Meles anakuma |
| NC_013276 | Mesocricetus auratus |
| NC_015241 | Microtus fortis fortis |
| NC_008064 | Microtus levis |
| NC_005035 | Mogera wogura |
| NC_006299 | Monodelphis domestica |
| NC_005279 | Monodon monoceros |
| NC_012694 | Moschus berezovskii |
| NC_004577 | Muntiacus crinifrons |
| NC_005089 | Mus musculus |
| NC_010650 | Mus terricolor |
| NC_011949 | Myrmecobius fasciatus |
| NC_006925 | Mystacina tuberculata |
| NC_010640 | Naemorhedus swinhoei |
| NC_005315 | Nannospalax ehrenbergi |
| NC_008216 | Nasalis larvatus |
| NC_008450 | Neofelis nebulosa |
| NC_014051 | Nomascus siki |
| NC_006522 | Notoryctes typhlops |
| NC_013700 | Nyctereutes procyonoides |
| NC_003033 | Ochotona collaris |
| NC_004029 | Odobenus rosmarus rosmarus |
| NC_014682 | Orcinus orca |
| NC_000891 | Ornithorhynchus anatinus |
| NC_002078 | Orycteropus afer |
| NC_012762 | Otolemur crassicaudatus |
| NC_001941 | Ovis aries |
| NC_001644 | Pan paniscus |
| NC_001643 | Pan troglodytes |
| NC_010641 | Panthera pardus |
| NC_007441 | Pantholops hodgsonii |
| NC_001992 | Papio hamadryas |
| NC_012103 | Pecari tajacu |
| NC_006521 | Perameles gunnii |
| NC_012764 | Perodicticus potto |
| NC_008830 | Phacochoerus africanus |
| NC_008137 | Phalanger vestitus |
| NC_008133 | Phascolarctos cinereus |
| NC_005280 | Phocoena phocoena |
| NC_002503 | Physeter catodon |
| NC_008219 | Piliocolobus badius |
| NC_005275 | Platanista minor |
| NC_002083 | Pongo abelii |
| NC_005277 | Pontoporia blainvillei |
| NC_006524 | Potorous tridactylus |
| NC_008217 | Presbytis melalophos |
| NC_014875 | Procapra przewalskii |
| NC_009126 | Procyon lotor |
| NC_013563 | Proedromys liangshanensis |
| NC_011053 | Propithecus coquereli |
| NC_006519 | Pseudocheirus peregrinus |
| NC_014698 | Pseudomys chapmani |
| NC_002612 | Pteropus dasymallus |
| NC_008431 | Pusa caspica |
| NC_008220 | Pygathrix nemaeus |
| NC_007703 | Rangifer tarandus |
| NC_001665 | Rattus norvegicus |
| NC_012374 | Rattus rattus |
| NC_014871 | Rattus sordidus |
| NC_012683 | Rhinoceros sondaicus |
| NC_011304 | Rhinolophus formosae |
| NC_005433 | Rhinolophus monoceros |
| NC_005829 | Rhyncholestes raphanurus |
| NC_007393 | Rousettus aegyptiacus |
| NC_012775 | Saimiri sciureus |
| NC_002369 | Sciurus vulgaris |
| NC_008215 | Semnopithecus entellus |
| NC_007631 | Sminthopsis crassicaudata |
| NC_010497 | Spilogale putorius |
| NC_012053 | Stenella coeruleoalba |
| NC_000845 | Sus scrofa |
| NC_003321 | Tachyglossus aculeatus |
| NC_004032 | Tamandua tetradactyla |
| NC_006518 | Tarsipes rostratus |
| NC_002811 | Tarsius bancanus |
| NC_002658 | Thryonomys swinderianus |
| NC_005825 | Thylamys elegans |
| NC_003039 | Trichosurus vulpecula |
| NC_013068 | Tscherskia triton |
| NC_002521 | Tupaia belangeri |
| NC_005034 | Urotrichus talpoides |
| NC_003426 | Ursus americanus |
| NC_012773 | Varecia variegata variegata |
| NC_003322 | Vombatus ursinus |
| NC_008434 | Vulpes vulpes |
| NC_006364 | Zaglossus bruijni |
